# Metabolic changes in response to varying whole-grain wheat and rye intake

**DOI:** 10.1038/s41538-024-00247-0

**Published:** 2024-01-30

**Authors:** Ville M. Koistinen, Sumanto Haldar, Marjo Tuomainen, Marko Lehtonen, Anton Klåvus, John Draper, Amanda Lloyd, Manfred Beckmann, Wendy Bal, Alastair B. Ross, Kirsten Brandt, Lee Fawcett, Chris Seal, Kati Hanhineva

**Affiliations:** 1 Food Sciences Unit, Department of Life Technologies, University of Turku, Turku, Finland; 2 Institute of Public Health and Clinical Nutrition, University of Eastern Finland, Kuopio, Finland; 3 Clinical Nutrition Research Centre, Singapore Institute of Food and Biotechnology Innovations (SIFBI), Yong Loo Lin School of Medicine, Singapore, 117599 Singapore; 4 School of Pharmacy, University of Eastern Finland, Kuopio, Finland; 5 Department of Biological, Environmental and Rural Sciences, Aberystwyth University, Wales, UK; 6 Human Nutrition and Exercise Research Centre, Population Health Sciences Institute, Newcastle University, Newcastle upon Tyne, UK; 7 AgResearch, Lincoln, New Zealand; 8 School of Mathematics, Statistics and Physics, Newcastle University, Newcastle upon Tyne, UK; 9 Food and Nutrition Science Division, Chalmers University of Technology, Gothenburg, Sweden

**Keywords:** Biomarkers, Metabolomics

## Abstract

Epidemiological studies have shown associations between whole-grain intake and lowered disease risk. A sufficient level of whole-grain intake to reach the health benefits has not been established, and there is limited knowledge about the impact of whole-grain intake on metabolite levels. In this clinical intervention study, we aimed to identify plasma and urine metabolites associated with two different intake levels of whole-grain wheat and rye and to correlate them with clinical plasma biomarkers. Healthy volunteers (*N* = 68) were divided into two groups receiving either whole-grain wheat or whole-grain rye in two four-week interventions with 48 and 96 g/d of whole grains consumed. The metabolomics of the plasma samples was performed with UPLC–QTOF-MS. Plasma alkylresorcinols were quantified with GC-MS and plasma and urinary mammalian lignans with HPLC-ECD. The high-dose intervention impacted the metabolite profile, including microbial metabolites, more in the rye-enriched diet compared with wheat. Among the increased metabolites were alkylresorcinol glucuronides, sinapyl alcohol, and pipecolic acid betaine, while the decreased metabolites included acylcarnitines and ether lipids. Plasma alkylresorcinols, urinary enterolactone, and total mammalian lignans reflected the study diets in a dose-dependent manner. Several key metabolites linked with whole-grain consumption and gut microbial metabolism increased in a linear manner between the two interventions. The results reveal that an increase in whole-grain intake, particularly rye, is strongly reflected in the metabolite profile, is correlated with clinical variables, and suggests that a diet rich in whole grains promotes the growth and/or metabolism of microbes producing potentially beneficial microbial metabolites.

## Introduction

Diets rich in whole-grain foods contribute to a lowered risk of several chronic diseases with high global disease burden, including type 2 diabetes, cardiovascular disease, coronary heart disease, stroke, and some cancers^1–4^. A lack of whole grains in the diet is reported to be the primary contributor to cardiovascular mortality and disability-adjusted life years and diets low in whole grains and fruit and high in sodium collectively accounted for more than half of diet-related deaths in 2017^5^. Pooled data across studies suggest dose-response relationships between whole-grain intake and all-cause mortality and disease risk^6–9^, but the mechanisms behind these effects are unclear. While some of these effects can be attributed to the high amount of dietary fiber present in whole grains relative to refined grains, another key mediator is the synergistic effect of a multitude of phytochemicals, bioactive plant-derived molecules, abundant in unrefined cereal products^10–12^. The phytochemicals in whole grains encompass several compound classes, including alkaloids, coumarins, phenolic acids, polyphenols, and terpenoids^13^, which may result in beneficial health effects through several potential mechanisms, including modulating cell differentiation and gene expression^10,14–16^.

Dietary recommendations for whole-grain intake exist in some countries, such as the United States^17^ and the Nordic countries^18^, and range from around 50 to 90 grams of whole grains per day depending on the energy intake. These recommendations have roots in ensuring sufficient dietary fiber intake and there is insufficient scientific evidence to establish an optimal level of whole-grain intake to achieve the disease risk reduction observed in epidemiological studies^19^. In recent years, a few studies have been published on the human plasma or serum metabolite profiles after the consumption of whole-grain rye compared with refined wheat but not as a direct comparison of different whole-grain cereals. Apart from known and potential molecular biomarkers of whole-grain intake, such as alkylresorcinols (AR) and mammalian lignans for wheat and rye^20–22^, avenanthramides and avenacosides for oats^23^ and recently suggested pipecolic acid betaine for rye^24^, the impact of different levels of whole-grain intake and from different cereals on the metabolic profile of human biofluids remains largely undefined^23^. In addition, most of the existing studies have included refined wheat as the control diet, and less is known about the potential differences between different whole grains, such as whole-grain wheat and rye.

The Human Metabolome Database currently (as of March 2022) contains 18593 metabolites detected—with or without quantification—in human blood plasma, of which 2451 originate from food, plants, or microbes^25^. Therefore, it is challenging from an analytical perspective to study samples which may contain a high number of different metabolites with wide chemical diversity and range of concentrations. Liquid chromatography–mass spectrometry (LC-MS) metabolomics has become a standard tool for investigating the metabolic changes occurring in nutritional studies, due to its high sensitivity, selectivity, and dynamic range^26^. However, LC-MS metabolomics is limited by its reliance on existing reference data to identify and annotate detected metabolites, many of which are still unidentified or lack unambiguous characterization.

This study aimed to investigate how two different levels and types of whole-grain intake impact the human plasma metabolome. The main objective was to determine the differential plasma metabolites related to two levels of whole-grain intake, at or double the typical intake recommendations (48 g and 96 g), and to compare markers of cardiometabolic disease risk between two distinct doses of whole-grain wheat and whole-grain rye intake. The secondary objectives were 1) to quantify known and potential new biomarkers of intakes of either whole-grain wheat or whole-grain rye, including plasma alkylresorcinols as well as plasma and urine mammalian lignans and assess their dose dependencies on the whole-grain intake levels and 2) to generate new hypotheses on the implications of the metabolic signature regarding the impact of whole-grain-rich diets on gut and overall health. Importantly, we aimed to compare responses to whole-grain wheat and whole-grain rye, which can elucidate potential differences between cereal species better than a comparison between whole-grain rye and white wheat, where the main difference is the presence or absence of the bran and germ fractions.

## Results

### Dietary intake

Energy and macronutrient intake were similar between various dietary intervention periods except for dietary fiber intake, which was significantly lower after the wash-out period (0 servings whole grains per day) compared with the volunteers’ habitual intake (measured at the Induction Visit). Energy intake was slightly higher in Dose 2 period compared with the washout period, the difference mainly due to an increase in carbohydrate intake from whole-grain foods. Mean daily fiber intake was significantly higher in Dose 1 (3 servings whole grains per day) and Dose 2 (6 servings whole grains per day) periods of the intervention compared with that during the wash-out period in both diet groups. There were also significant increases in thiamine, iron, manganese, magnesium, phosphorus and zinc intake with increasing whole-grain intake in both whole-grain wheat (WGW) and whole-grain rye (WGR) groups compared with that during the Dose 0 period; intake of riboflavin in the WGW group and that of vitamin E in the WGR group also increased with increasing doses of whole-grain intake (all *p* < 0.05; Supplementary Table 1).

### Whole grain and alkylresorcinol intake

The reported amounts of whole-grain intakes of both diet groups were similar at various stages of dietary intervention (Table 1). Whole-grain intakes were significantly reduced during the Dose 0 period compared with their intake at the beginning of the study (Induction Visit), indicating good reported compliance to the dietary intervention regime. Similarly, reported whole-grain intakes at Dose 2 period for both diet groups were approximately two times that in Dose 1 period (), but was slightly higher than planned (96 g whole grains per day). The calculated mean daily intake of the respective alkylresorcinol homologs in the WGW and WGR groups increased in a similar manner (Supplementary Table 2). Intake of AR C17:0 in the WGR group was significantly higher during both Dose 1 and Dose 2 periods compared with their respective intakes for the WGW group, reflecting the difference in alkylresorcinol homolog distribution between wheat and rye^27^.

**Table 1:** Reported total whole-grain intake for whole-grain wheat (WGW) and whole-grain rye (WGR) groups during the study, using Food Frequency Questionnaires (FFQ).

Group	Induction Visit		Wash-out (Dose 0)		Dose 1		Dose 2	
	Mean (g/d)	SD	Mean (g/d)	SD	Mean (g/d)	SD	Mean (g/d)	SD
WGW	52.8	39.65	0.9	3.12	58.1	29.91	127.0	59.87
WGR	55.3	34.29	1.5	4.78	57.8	39.14	114.3	54.53

Wash-out (Dose 0) was a 4 wk whole grain avoidance diet, Dose 1 required 48 g/d whole grain intake for 4 weeks and Dose 2 required 96 g/d whole grain intake for a further 4 weeks.

### Impact of whole grains on the metabolite profile

A principal component analysis (PCA) was performed separately for all detected good-quality molecular features (*n* = 6629; see Data analysis and statistics for determining the good-quality features) and only those good-quality features which had a statistically significant change in at least one of the statistical tests (*q* < 0.1; *n* = 1152) (Supplementary Fig. 1). While the interventions with increasing whole-grain intake impacted the metabolite profile of individual volunteers, no clear separation or trend was seen in the direction of the change in either the WGW or the WGR group even when including only differential metabolites.

**Fig. 1:**
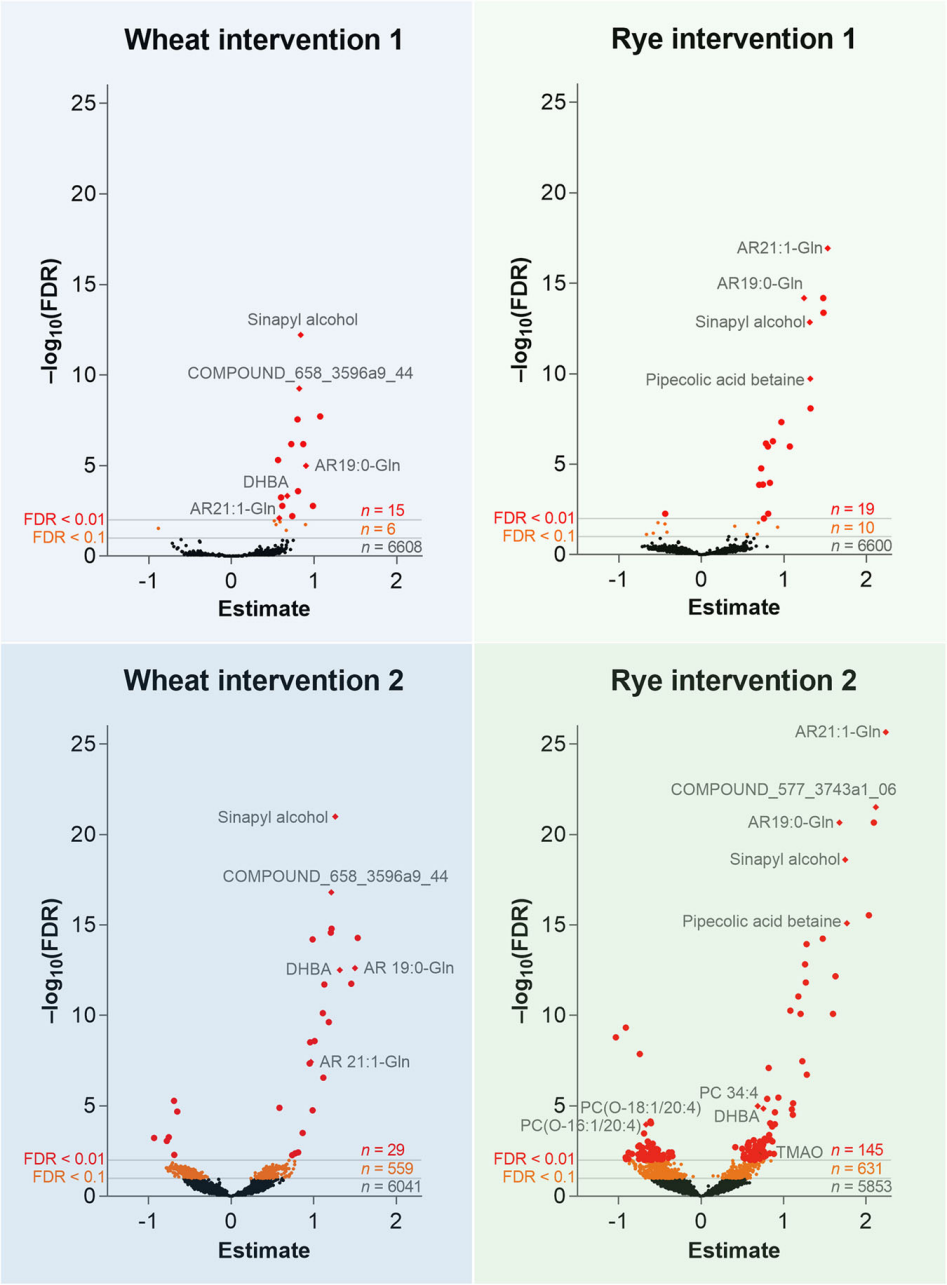
Volcano plots of the metabolic impact. The volcano plots show the effect of each intervention on the good-quality metabolite features (*n* = 6629). Annotations are included for the metabolites with highest and most significant changes. The estimate is the regression coefficient of the linear mixed model and reflects the increase (positive value) or decrease (negative value) of the metabolite compared to baseline.

**Fig. 2:**
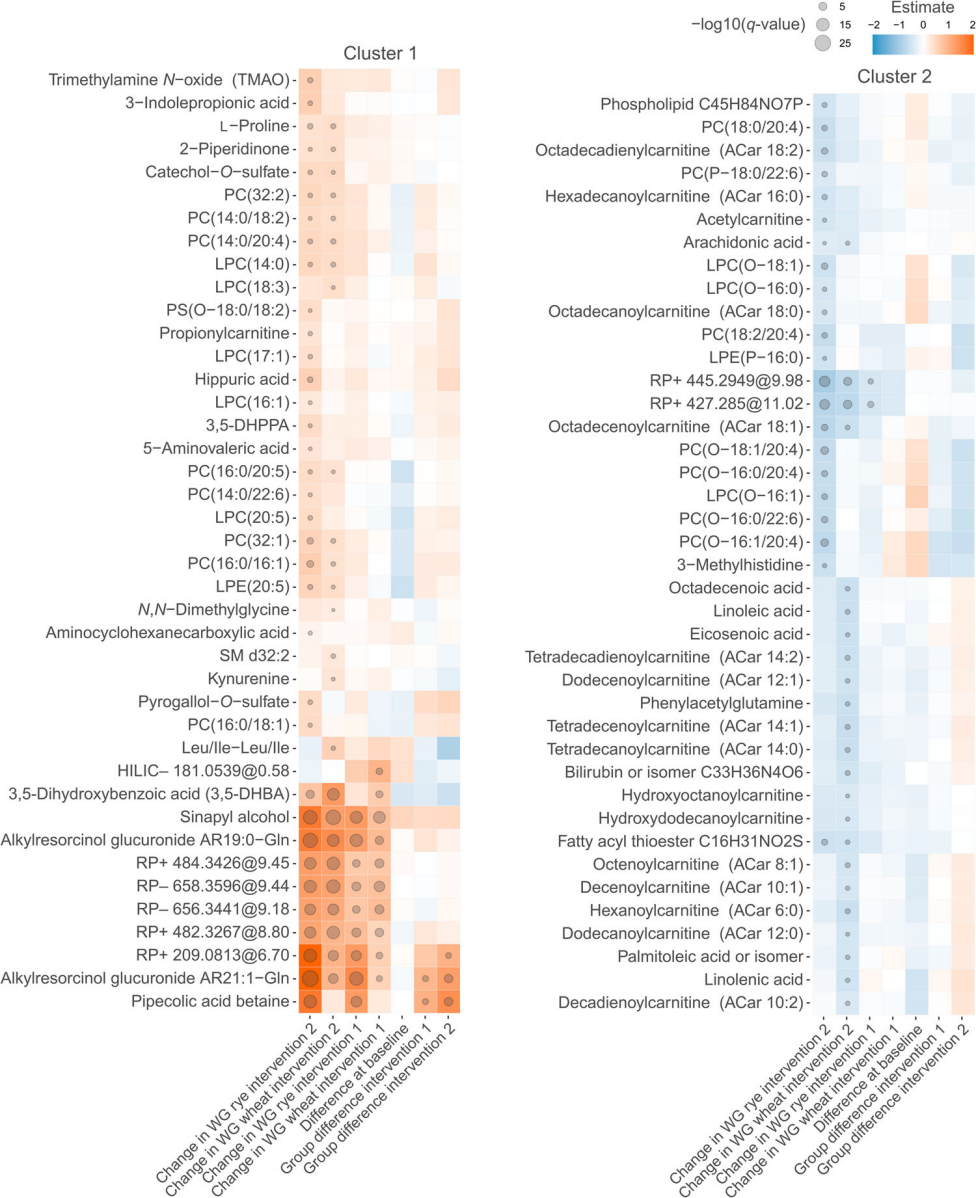
Heat map of the differential metabolites. The compounds (*n* = 81) include eight most differential unidentified molecular features, in at least one of the six comparisons in the linear mixed model, including change in the wheat or rye interventions and difference between the wheat and rye groups after each intervention. Differences with *q* < 0.1 are marked with a circle. The estimate is the standardized regression coefficient from the statistical model; in the group difference comparisons, a higher estimate value represents increase of the metabolite in rye group compared to wheat. One identified metabolite (tryptophan betaine) had a significant difference between the two groups at baseline and was discarded from further analysis (data not shown).

**Fig. 3:**
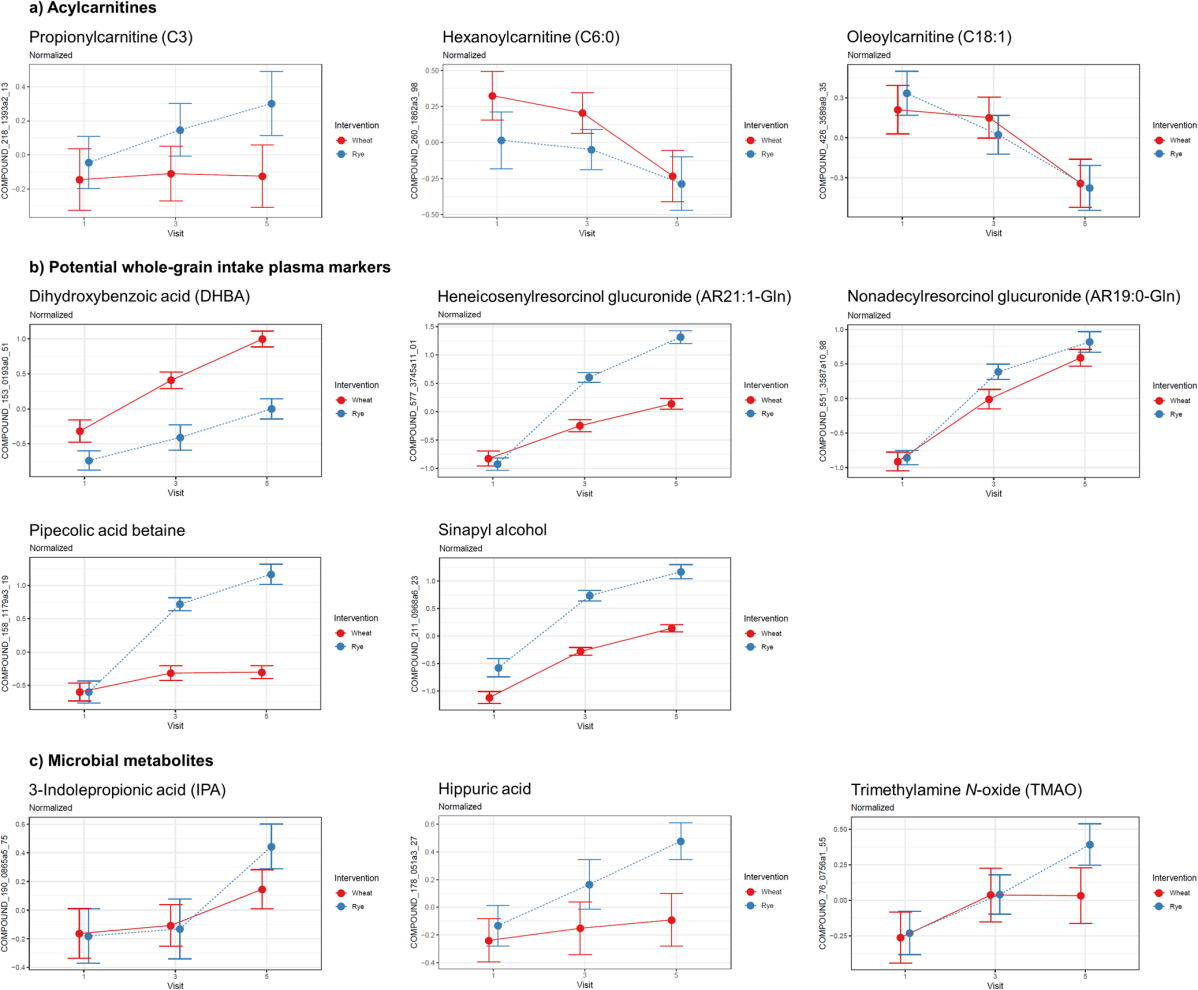
Line plots of differential metabolites. **a** Acylcarnitines, **b** potential plasma biomarkers of whole-grain intake, and **c** microbial metabolites with their normalized levels at baseline (visit 1), at the end of the first intervention (visit 3), and at the end of the second intervention (visit 5). The error bars represent one standard error of the mean (s.e.m).

At the end of the Dose 1 period with 3 servings of whole grains per day, wheat intake had caused a statistically significant change (*q* < 0.1) in 21 molecular features and rye intake in 29 molecular features. After the Dose 2 period with 6 servings of whole grain per day, wheat intake caused a change in 588 molecular features and rye intake in 776 molecular features compared to baseline (*q* < 0.1), out of which 29 and 145 had *q* < 0.01 in WGW and WGR, respectively (Fig. 1, Supplementary Fig. 2). Altogether, 73 differential metabolites were identified or putatively annotated, and they formed two distinct clusters in the hierarchical clustering analysis based on their increase or decrease during the intervention (Fig. 2). Among the increased metabolites in both whole-grain study groups and both Doses were two alkylresorcinol glucuronides, heneicosenylresorcinol glucuronide (AR C21:1-Gln) and nonadecylresorcinol glucuronide (AR C19:0-Gln), as well as sinapyl alcohol (Fig. 2). Other molecular features that increased in both study groups (but not all Doses) were identified as, 3,5-dihydroxybenzoic acid (3,5-DHBA), catechol-*O*-sulfate, 2-piperidinone, proline, and various phospholipids. Pipecolic betaine increased only in the WGR group during both Doses. Metabolites only increasing during the Dose 2 phase of WGR groups include trimethylamine *N*-oxide (TMAO), 3-(3,5-dihydroxyphenyl)-1-propanoic acid (3,5-DHPPA), 5-aminovaleric acid, hippuric acid, 3-indolepropionic acid, and propionylcarnitine (Figs. 2 and 3).

The higher WGR intervention dose induced a decrease in several plasmenyl-type ether phospholipids, including LPC(O-16:1), LPC(O-16:0), LPC(O-18:1), PC(O-16:1/20:4), PC(O-16:0/20:4), PC(O-16:0/22:6), and PC(O-18:1/20:4), all of which were detected in the RP − /RP+ mode. They were annotated based on the characteristic MS/MS fragments of *m/z* 184 in the positive mode (for PCs) or *m/z* 168 in the negative mode (for LPCs). MS-FINDER^28^ was used to calculate the molecular formula, which was then transferred to LIPID MAPS^29^ to search for the identity of the metabolite and to further compare MS/MS reference spectra. The WGW Dose 2, in contrast to the corresponding WGR dose, caused a decrease in several medium- and long-chain acylcarnitine species, namely hexanoylcarnitine (ACar 6:0), octenoylcarnitine (ACar 8:1), decenoylcarnitine (ACar 10:1), decadienoylcarnitine (ACar 10:2), dodecanoylcarnitine (ACar 12:0), dodecenoylcarnitine (ACar 12:1), tetradecanoylcarnitine (ACar 14:0), tetradecenoylcarnitine (ACar 14:1), and tetradecadienoylcarnitine (ACar 14:2). These molecules were annotated based on a characteristic MS/MS fragment in the RP+ mode at *m/z* 85.029 and matching entries in our in-house and public spectral databases. Due to the untargeted nature of the analysis, the location of the double bonds in the acyl chains could not be determined. Acetylcarnitine and certain long-chain acylcarnitines, including hexadecanoylcarnitine (ACar 16:0) and octadecadienylcarnitine (ACar 18:2), only decreased during Dose 2 compared with baseline. Octadecenoylcarnitine (ACar 18:1) decreased during the Dose 2 period in both cereal groups.

### Plasma alkylresorcinol concentrations

Plasma concentrations of all alkylresorcinol homologs increased significantly on both Dose 1 and Dose 2 of the WGW and WGR diets compared to that during Dose 0 (Supplementary Fig. 3). The intra-class correlation coefficients (ICC) within paired visits (as separated by two days) for each dose of whole grains for all plasma alkylresorcinol homologs were moderate in both WGR and WGW groups ranging from an average ICC (for both groups combined) of 0.54 for AR C17:0 to about 0.74 for AR C19:0. Due to the different profiles of alkylresorcinol homologs present in WGR compared with WGW, the plasma C17:0 to C21:0 ratio was significantly different between the two diet groups at the end of Dose 1 and Dose 2 periods, although the ratio remained constant within each diet group, irrespective of the dose of whole grains (Supplementary Fig. 4).

LME models adjusted for age, gender and BMI were used to investigate the dose-response trends between whole-grain intake throughout the study and plasma alkylresorcinol concentrations. There were significant positive linear dose-response trends between WGR or WGW intake and all individual alkylresorcinol homologs and total alkylresorcinols (all *p* < 0.0001; Table 2). The Pearson correlation coefficients (*R*) for the dose-response trend for all alkylresorcinols were greater in the WGW group than the WGR group, even though the actual increase in plasma concentrations of alkylresorcinol were greater in the WGR group. Similarly, there were significant positive linear dose-response trends between the mean alkylresorcinol intake of the individual homologs during Dose 1 and Dose 2 periods of dietary intervention separately and their corresponding alkylresorcinol concentration in plasma (all *p* < 0.0001; data not shown).

**Table 2:** Dose-response trends between WG intake against measured plasma AR homolog and total AR concentrations in WGW and WGR groups, using linear mixed effect models, adjusted for age, gender and BMI.

	WGW Group					WGR Group				
Homolog	Slope (ln)^a^	R	*p-*value	Constant	Regression equation	Slope (ln)	R	*p-v*alue	Constant	Regression equation
AR C17:0	+0.015	0.673	<0.0001	1.252	1.25 + 0.015WG	+0.006	0.693	<0.0001	0.383	0.38 + 0.006WG
AR C19:0	+0.015	0.660	<0.0001	2.337	2.34 + 0.015WG	+0.015	0.760	<0.0001	2.039	2.04 + 0.015WG
AR C21:0	+0.012	0.637	<0.0001	2.633	2.63 + 0.012WG	+0.015	0.779	<0.0001	2.506	2.51 + 0.015WG
AR C23:0	+0.014	0.657	<0.0001	1.812	1.81 + 0.014WG	+0.013	0.793	<0.0001	1.527	1.53 + 0.013WG
AR C25:0	+0.018	0.646	<0.0001	1.550	1.55 + 0.018WG	+0.013	0.719	<0.0001	0.925	0.92 + 0.013WG
Total AR	+0.015	0.666	<0.0001	3.629	3.63 + 0.015WG	+0.015	0.782	<0.0001	3.308	3.31 + 0.015WG

The slope is the mixed effect of the WG in g/day.

^a^Natural logarithm.

### Urinary and plasma mammalian lignan concentrations

In contrast to the plasma alkylresorcinol measurements, there were limited significant differences in plasma mammalian lignan concentrations between various doses of whole-grain intake. A weak difference emerged in plasma enterolactone concentration between Dose 0 and Dose 2 (*p* < 0.05, Supplementary Fig. 5). Moreover, variation in plasma mammalian lignan concentrations was nearly as great within the paired visit measurements for each dose as between the increasing whole-grain doses (Supplementary Fig. 5), particularly in the WGW group and this variability was greater for enterodiol than for enterolactone. However, the differences in 24-h urinary total mammalian lignan excretion between various doses of whole-grain intake were more significant than plasma mammalian lignan concentrations, particularly between Dose 0 and Dose 2 intakes (Supplementary Fig. 6). Since 24-h urine samples were collected only once after each phase of dietary intervention, it was not possible to determine the repeatability of paired sampling, as done for plasma. The concentrations of the two mammalian lignans in urine were much greater (more than 50-fold) than those in plasma.

Unlike for plasma alkylresorcinols, there were no significant (linear or otherwise) dose-response trends between mean WGW intake and plasma mammalian lignans, although mean WGR food intake had a weak positive association with plasma enterolactone but not with enterodiol or total mammalian lignans in plasma (Supplementary Table 3, *p* = 0.03). However, there were significant positive linear dose-response trends between WGR and WGW intake and 24-h urinary excretion of enterolactone and total mammalian lignans (all *p* < 0.01), although there was no association with urinary enterodiol concentration/excretion (Supplementary Table 3).

### Clinical biomarkers and their relationship with plasma AR and metabolites

Anthropometric measures along with markers of cardiometabolic risk taken at recruitment and after each phase of the intervention study are shown in Table 3. Plasma samples were not collected at the induction visit and were available only for post wash-out, dose 1 and dose 2 timepoints. The fasting plasma concentrations of clinical biomarkers were not significantly affected by either of the whole-grain interventions and between the lower dose and higher dose of either cereal.

**Table 3:** Mean and standard deviation (SD) of anthropometric and cardiometabolic measures during each stage of the intervention study.

	Whole-Grain Wheat Group								Whole-Grain Rye Group							
	Induction Visit		Wash-out		Dose 1		Dose 2		Induction Visit		Wash-out		Dose 1		Dose 2	
	Mean	SD	Mean	SD	Mean	SD	Mean	SD	Mean	SD	Mean	SD	Mean	SD	Mean	SD
Measurement																
BMI^a^ (kg/m^2^)	25.6	3.27	25.4	3.17	25.4	3.21	25.5	3.25	26.1	3.27	25.8	3.22	25.9	3.18	26.0	3.20
Body fat (%)	28.3	7.55	29.1	8.02	28.7	7.62	28.8	7.54	29.2	6.67	29.4	6.37	29.0	6.43	29.7	6.55
Waist (cm)	87.3	11.85	87.0	11.04	87.2	11.11	87.5	10.99	88.2	13.81	88.4	12.73	88.0	12.51	88.8	12.76
SBP^b^ (mmHg)	128.0	22.14	126.5	18.51	124.7	16.64	123.7	15.22	129.5	16.53	127.6	18.26	126.7	14.85	123.4	13.86
DBP^c^ (mmHg)	77.7	12.06	77.3	10.72	76.9	9.92	75.5	9.18	77.8	9.43	75.5	9.23	76.4	9.08	73.7	8.88
Metabolic markers:																
Total Cholesterol (mM)			5.86	0.18	5.83	1.06	5.75	0.95			6.11	0.85	5.97	0.94	5.88	0.85
LDL Cholesterol (mM)			3.58	0.96	3.53	0.95	3.43	0.83			3.86	0.82	3.71	0.85	3.60	0.75
HDL Cholesterol (mM)			1.68	0.39	1.69	0.40	1.70	0.43			1.68	0.38	1.63	0.36	1.65	0.39
Triglycerides (mM)			1.35	0.78	1.36	0.68	1.35	0.66			1.25	0.60	1.40	0.66	1.38	0.68
Glucose (mM)			4.96	0.30	5.12	0.45	4.89	0.33			5.08	0.41	4.78	0.31	4.95	0.38
Insulin (IU/mL)			5.95	2.99	5.96	3.08	6.12	3.34			5.97	4.02	5.83	3.58	6.57	4.24
HOMA-IR			1.54	0.99	1.39	0.93	1.56	1.01			1.15	0.64	1.22	0.56	1.23	0.74
CRP (mg/dL)			2.38	3.79	1.88	2.07	2.29	4.36			1.20	1.09	2.40	5.89	1.64	1.57
e-selectin (ng/mL)			20.66	8.95	20.40	8.04	20.58	9.80			23.58	11.72	22.39	11.32	24.12	11.20
ICAM (ng/mL)			117.61	31.10	120.63	34.42	121.92	36.81			115.28	34.26	107.11	30.20	108.66	26.24
VCAM (ng/mL)			174.10	39.76	179.33	43.54	178.18	52.58			186.75	61.86	180.81	76.23	181.04	68.01
Homocysteine (µM)			12.12	2.69	12.44	2.49	12.04	2.48			12.67	4.83	12.78	3.77	13.08	3.83
Glycerol (µM)			111.44	64.84	119.98	60.29	99.30	50.39			107.03	67.51	100.21	52.14	84.23	38.43
FRAP (Fe^3+^ ion equivalents)			243.2	54.07	244.4	52.65	242.4	47.32			243.3	39.19	235.8	36.06	235.8	32.75

^a^BMI, Body mass index.

^b^SBP, systolic blood pressure.

^c^DBP, diastolic blood pressure.

Using LME models, the associations between reported whole-grain intake using FFQ, biomarkers of whole-grain intake (plasma total alkylresorcinols, plasma and urine mammalian lignans) and markers of cardiometabolic disease risk were compared with reported whole-grain intake using FFQ. The data are presented in Table 4. For the pooled group, significant negative associations of plasma alkylresorcinols with total cholesterol and LDL cholesterol were greater than the associations with either reported whole-grain intake or with urinary mammalian lignan excretion. The negative associations with systolic BP were significant with plasma alkylresorcinols but not with the other targeted measurements. The significant negative association of fasting plasma glucose with reported whole-grain intake was marginally greater with reported whole-grain intake than with plasma alkylresorcinol concentration, although no such association was found for mammalian lignan concentrations.

**Table 4:** Associations of whole-grain intake, plasma AR, plasma lignans, and urinary lignans with markers of cardiometabolic disease risk from linear mixed-effect models.

Variable	Reported WG intake	Plasma total alkylresorcinols	Plasma total lignans	Urine total lignans
Systolic BP	−ve (marginal; *p* = 0.07)	−ve (*p* < 0.05)‡	NS	NS
Diastolic BP	NS	NS	NS	NS
BMI	+ve (*p* < 0.01)	+ve (*p* < 0.001)*	NS	+ve (*p* < 0.01)‡
Total chol	−ve (*p* < 0.05)*	−ve (*p* < 0.01)*	NS	−ve (*p* < 0.05)†
LDL chol	−ve (marginal; *p* = 0.06)	−ve (*p* = 0.001)*	NS	−ve (*p* < 0.05)†
HDL chol	NS	NS	NS	NS
TG	+ve (marginal p = 0.06)*	NS	NS	NS
Glucose	−ve (*p* < 0.001)‡	−ve (*p* < 0.005)†	NS	NS
Insulin	NS	NS	NS	NS
Log(HOMA-IR)	NS	NS	NS	NS
Log(CRP)	+ve (marginal; *p* = 0.08)*	+ve (marginal; *p* = 0.08)*	NS	NS
E-selectin	NS	NS	NS	NS
ICAM-1	NS	NS	NS	NS
Log(Homocysteine)	+ve (marginal; *p* = 0.09)*	+ve (marginal; *p* = 0.07)*	NS	NS
Log(Glycerol)	NS	NS	NS	NS
Log(FRAP)	−ve (marginal; *p* = 0.07)*	NS	NS	NS

Effect driven mainly by: * WGR group; † WGW group; ‡ both groups.

**Fig. 4:**
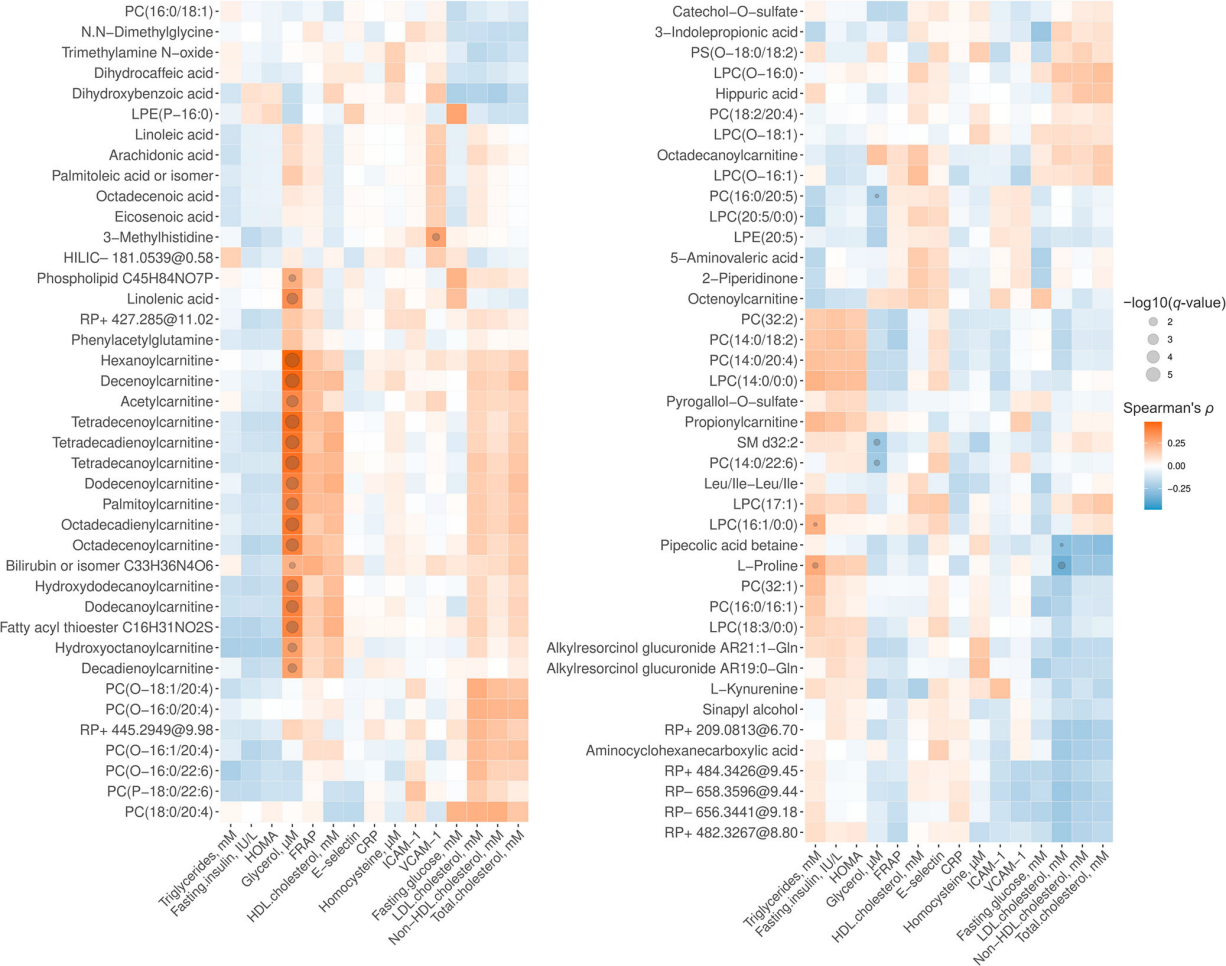
Heat map of correlations between differential metabolites and clinical markers. The Spearman correlations were calculated between the differential metabolites (*n* = 81) from the intervention and 15 clinical markers from plasma. Correlations with *q* < 0.1 are marked with a circle.

To study the potential linkage between metabolite alterations evoked by the whole-grain-rich diets and the clinical status of the participant, we calculated the Spearman correlations between the significantly altered metabolites and 14 fasting plasma markers and HOMA visualized as a heatmap in Fig. 4. The strongest positive correlations were observed between glycerol and most of the annotated acylcarnitines and a few other individual lipids, including the 2:0, 6:0, 8:0-OH, 10:1, 10:2, 12:0, 12:0-OH, 12:1, 14:0, 14:1, 14:2, 16:0, 18:1, and 18:2 ACar species, all of which decreased during the Dose 2 intervention of rye and/or wheat. 3-Methylhistidine was positively correlated with vascular cell adhesion molecule-1 (VCAM-1) (*ρ* = 0.29, *q* = 0.026). l-Proline correlated positively with triglycerides (*ρ* = 0.27, *q* = 0.060) and inversely with LDL cholesterol (*ρ* = −0.35, *q* = 0.029). Pipecolic acid betaine was inversely correlated with LDL cholesterol (*ρ* = −0.29, *q* = 0.098).

## Discussion

The overall metabolite profile was impacted more by the higher whole-grain intervention compared with the lower intakes, both in terms of the number of significantly changed features and the magnitude of the observed change (Fig. 1). An increase was observed in the concentrations of several metabolites, corresponding with the increasing amount of whole grains ingested during the study (Fig. 3), which suggests that the concentration of many metabolites co-vary with whole-grain intake, which may reflect some of the biological effects associated with wholegrain intake at higher doses. However, the shape of the dose-response relationship cannot be reliably assessed due to the limited number of measurements with different whole-grain intakes. In a series of meta-analyses of prospective studies, Schwingshackl and colleagues reported that increased whole-grain intake was associated with reduced all-cause mortality^9^ and colorectal cancer^30^. The reduction of all-cause mortality and colorectal cancer was linear between 0 to at least 100 g of daily whole grain consumption, which covers the amounts consumed in this study (0, 48, and 96 g/day). In the case of type 2 diabetes, the risk reduction is linear until about two servings per day^8^. The compounds revealed by our non-targeted metabolomics approach may contribute to the molecular mechanisms behind the demonstrated risk reduction. The lack of a visible trend in the overall metabolite profiles of the volunteers in the PCA (Supplementary Fig. 1) is typical when studying human subjects, due to the multitude of variables affecting the metabolism and inter-individual variability in the metabolic responses to the intervention. Nevertheless, some of the individual metabolite profiles changed markedly during the intervention when examined by the two components representing highest variability in the dataset. There is considerable interest in understanding these inter-individual differences in response to nutritional intervention studies, and these divergent results in metabolite profiles, in a well-controlled design, although resulted in clear trends for specific biomarkers, support the suggestion that there should be more attention paid to understanding inter-individual variation in response to whole grains.

The metabolites which showed the highest increases included several compounds originating from the whole grains themselves: two alkylresorcinol glucuronides, dihydrobenzoic acid, pipecolic acid betaine, and sinapyl alcohol. The two alkylresorcinols, glucuronidated in the liver, were reported by Hanhineva et al. as the two main plasma biomarker candidates for whole-grain intake^31^. Based on the results in the current study, AR C19:0-Gln similarly reflected both whole-grain whreat (WGW) and whole-grain rye (WGR) intake, while AR C21:1-Gln was increased about twice as much by the WGR diet compared with WGW in both interventions. This roughly corresponds with the proportions of the alkylresorcinol precursors, AR C19:0 and AR C21:0, in intact wheat and rye grains^32^. Interestingly, in contrast to the high abundance of AR C21:0 compared with AR C21:1 in whole grains, we did not observe any major peak for AR C21:0-Gln in our data, which corresponds with the previous findings^31^. 3,5-DHBA and 3,5-DHPPA, which were increased by the intervention diets, are considered endogenous metabolites of alkylresorcinols and also proposed as biomarkers for the intake of alkylresorcinol-containing foods, mainly whole-grain wheat and rye^33^. Interestingly, 3,5-DHBA increased more in the WGW intervention group compared with WGR, whereas 3,5-DHPPA only increased significantly in the WGR intervention group, which can be explained by the different alkylresorcinol homologs profile producing a different proportion of these two metabolites, but not by the total alkylresorcinol content, which was not significantly different between WGW and WGR intervention^34^. This difference may also be explained by the relatively high proportion of unsaturated alkylresorcinol homologs in rye relative to wheat (15–20% compared with <5%), and that 3,5-DHBA is likely one of the final metabolites in mammalian alkylresorcinol metabolism. The finding that many of the more important metabolites that are associated with the whole-grain interventions were related to alkylresorcinols confirms that this class of compounds are well suited to be biomarkers for wheat and rye intake.

We have previously proposed pipecolic acid betaine as a biomarker of the Healthy Nordic Diet, which is rich in whole grains, and particularly the intake of WGR^24,35,36^. The results presented in the present study confirm that pipecolic acid betaine differentiates between wheat and rye diets, as its concentrations increased significantly in the WGR group but not in the WGW group. This compound has been detected in rye flour but not in any other cereal or pseudocereal product^37^, which makes it a potential candidate for (whole-grain) rye intake. In this study, pipecolic acid betaine was also inversely correlated with fasting insulin, which is in line with our previous findings^24^ and likely reflects the previously observed insulin-lowering effect associated with increased rye intake, also known as the ‘rye factor’^38^.

Sinapyl alcohol is one of the three components of lignin, a structural polymer in plants (the two others being *p*-coumaryl and coniferyl alcohol); Bunzel et al. showed the existence of authentic lignin in cereal bran by chemically cleaving polymers within the bran matrix and detecting coniferyl and sinapyl alcohol as the liberated monolignols^39^. Sinapyl alcohol has shown anti-inflammatory and antinociceptive effects in a mouse model^40^. We inspected our existing untargeted metabolomics data from unprocessed WGR and WGW bread samples and did not find detectable levels of sinapyl alcohol (data not shown). Therefore, it is likely that the sinapyl alcohol detected in the plasma originates from the gut microbial degradation of cereal lignin, because humans lack endogenous enzymes to cleave lignin, and it is also highly resistant to acid- or base-catalyzed hydrolysis. Sinapyl alcohol could, therefore, be suggested as a potential biomarker candidate for lignin-based fiber intake, although its source remains to be confirmed.

Propionylcarnitine, a short-chain acylcarnitine, is produced in the liver from propionyl-CoA, which may originate from several sources, including amino acids (valine, methionine, threonine, and isoleucine), odd-chain fatty acids, or short-chain fatty acids (SCFA)^41,42^; however, it is thought that propionic acid produced by colonic microbiota is the main source of propionyl-CoA^41^. Propionic acid itself is a product of the microbial fermentation of dietary carbohydrates, mainly resistant starch, and to lesser extent of proteins and amino acids^43^. The increase in propionylcarnitine levels in the WGR group (but not in WGW) thus strongly suggests that microbial SCFA metabolism was increased due to a higher supply of fermentable substrate in the diet. In the long term, the dietary change may have also supported the growth of microbial strains most capable of producing SCFAs. Due to their poor ionization efficiency, SCFAs themselves were outside of the analytical range of the LC-MS instrument, and therefore were not detected in this study.

**Fig. 5:**
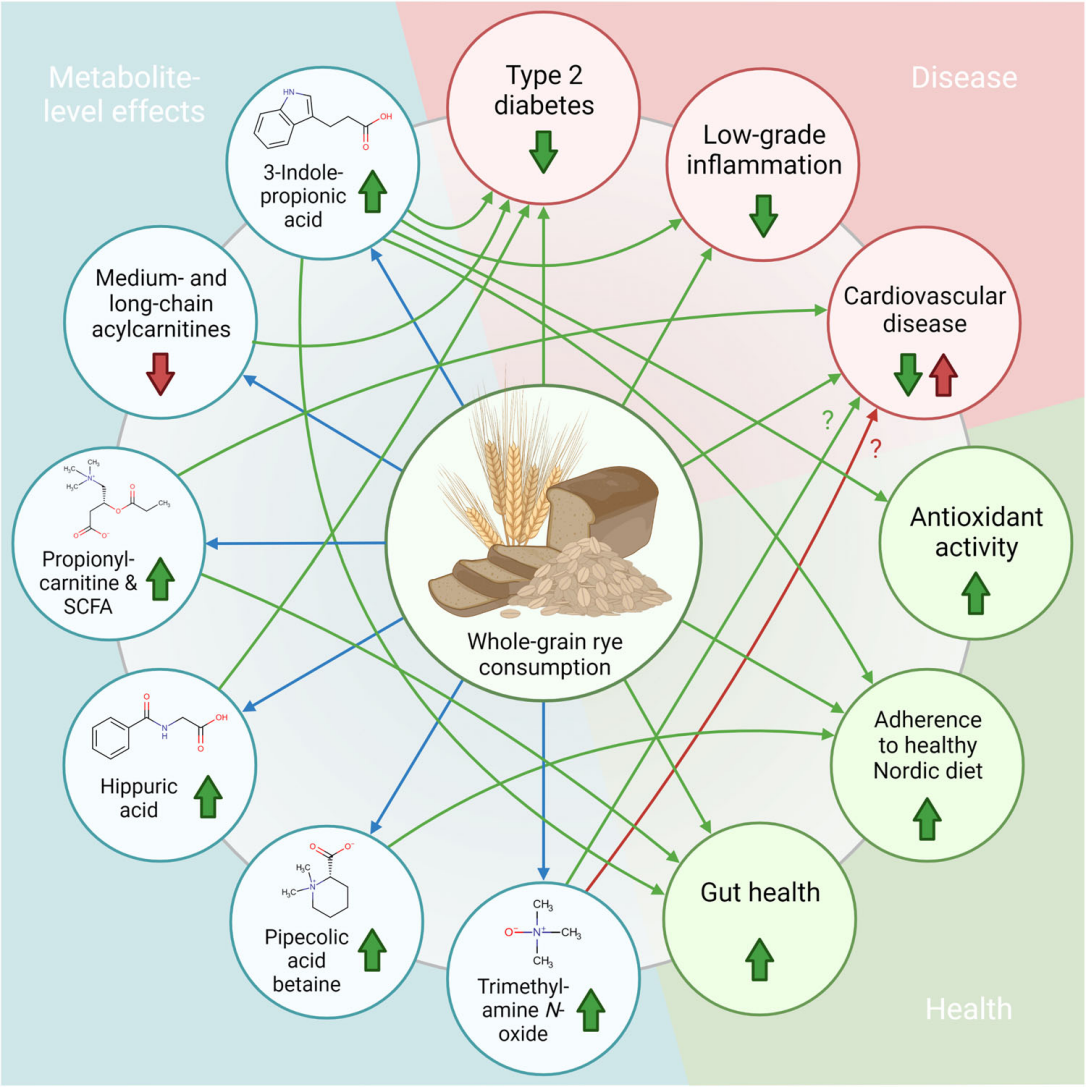
Known associations between whole-grain rye consumption, health and disease, and metabolites increased or decreased by rye intake in the current study. The associations involving the metabolites reported in the current study are marked with blue arrows, other known associations with potential health benefit with green arrows, and associations potentially harmful with red arrows.

3-Indolepropionic acid, a gut microbial metabolite of tryptophan, has been associated with a lower type 2 diabetes risk^44^, reduced low-grade inflammation^45^, rye and dietary fiber intake^44,45^, and the Healthy Nordic Diet^46^ (Fig. 5). In the current study, the increase of 3-indolepropionic acid in the WGR intervention is consistent with the previous observations by ref. ^44^; in addition, this study confirms that the increase is not only dependent on the whole-grain content of the diet but also on the cereal species, since the level of the metabolite did not significantly increase during the WGW intervention. In the current study, however, 3-indolepropionic acid was weakly associated with the fasting plasma glucose concentrations, although not significantly after false discovery rate correction, and no association was found with fasting plasma insulin concentrations (Fig. 4). This may be because the insulin-lowering effect of rye is most evident in postprandial plasma^47^. Whereas the increased SCFA production tentatively associated with rye intake in this study could be explained by the increased amounts of polyfructan precursors in rye. The increase of 3-indolepropionic acid only in the WGR intervention cannot be attributable to tryptophan content, because wheat has a higher content of free and protein-bound tryptophan compared with rye^48^. Thus, we can hypothesize that rye consumption instead promotes the proliferation of 3-indolepropionic acid–producing gut bacteria.

Similar to 3-indolepropionic acid, trimethylamine *N*-oxide (TMAO) was significantly higher than the baseline level only after the higher WGR intervention. It is an oxidized liver metabolite of trimethylamine, which in turn is a gut microbial metabolite of several dietary molecules, including glycine betaine, carnitine, and choline^49^. TMAO has been a subject of controversy in recent years because it has been associated with both increased and decreased risk of atherosclerosis and cardiovascular disease, and furthermore, some dietary sources associated with reduced cardiovascular disease risk, such as marine fish, have high concentrations of bioavailable TMAO^49,50^. Whole grains are another food group associated with decreased cardiovascular disease risk^50,51^, and the increase in TMAO levels during the WGR intervention may suggest that TMAO is an indicator of increased gut microbial metabolic activity and a bystander, rather than mediator, in cardiovascular disease development. In our earlier work with subjects from the Nordic countries, TMAO was not associated with dietary fiber intake or other rye biomarkers, such as pipecolic acid betaine^24^, which may suggest that individual variability in gut microbiota may play a role in how TMAO correlates with clinical and dietary markers.

Hippuric acid is the metabolic end product of a variety of compounds, most notably proteins and dietary polyphenols from plant foods^52^. As for TMAO, hippuric acid is a host–microbial co-metabolite, a liver conjugate of glycine and benzoic acid. The benzoic acid moiety originates from the microbial degradation of aromatic amino acids, phenolic acids, flavonoids, and other aromatic compounds, including some toxicants. In a bilberry intervention study by de Mello et al., hippuric acid was associated with improved glucose and insulin metabolism in individuals at high risk of type 2 diabetes^52^. Although WGR is a rich source of phenolic acids, this does not explain why only the WGR-enriched diet caused an increase in the hippuric acid concentration, given that WGW also contains many of the same phenolic acids, and other precursors would be present in the non-cereal part of the study diets. The most plausible explanation, as with 3-indolepropionic acid and TMAO, is that the high amount of microbiota-accessible carbohydrates in rye relative to WGW boosts the growth of certain microbes and results in a higher abundance of these metabolites in plasma of individuals who have consumed high amounts of WGR.

Increased concentrations of medium- and long-chain acylcarnitines are one sign of impaired fatty acid oxidation in mitochondria, which contributes to insulin resistance^53^. We found that the concentrations of acylcarnitines with hydrocarbon chains C6:0, C12:0, C16:0, C18:1, and C18:2 were decreased in plasma during the second intervention with the six daily servings of WGW or WGR products (Figs. 2 and 3). The decrease was observed in both groups receiving wheat or rye for C18:1 (octadecenoylcarnitine), while C16:0 and C18:2 were significantly decreased only in the group receiving WGR and C6:0 and C12:0 concentrations were decreased only in the WGW group. Thus, the acylcarnitine concentrations may reflect lower circulating lipid levels for the corresponding fatty acids as well as indicate that the whole-grain intervention had a beneficial effect on the mitochondrial energy metabolism, which could further contribute to the lowered risk of insulin resistance and type 2 diabetes, already strongly associated with whole-grain intake in epidemiological studies^54^. Although not a main focus of this study, we observed a consistent positive relationship between plasma acylcarnitines and plasma glycerol. This suggests a relationship between the release of fatty acids from triglycerides and fatty acid oxidation during the intervention study. We have previously reported that 5-aminovaleric acid betaine, a metabolite associated with whole-grain intake, decreases the β-oxidation of fatty acids in mouse heart tissue and may improve cardiac function during ischemic conditions^55^. Thus, this study provides further evidence of the potential cardioprotective effects of whole grains.

The concentrations of all six measured alkylresorcinol homologs in plasma reflected the reported whole-grain intake from the FFQ data in a dose-dependent manner. Alkylresorcinol C17 and C21 ratios in plasma correlated with the WGW and WGR intake, aligned with their relative concentrations in wheat and rye^33^. Alkylresorcinol concentrations showed no significant differences between paired visits, confirming single blood samples suffice for stable whole-grain diet representation, distinguishing WGW and WGR intake^56^. However, the short half-life of alkylresorcinols limits the generalization of the results to only populations with stable whole-grain intake^57^. These results confirm plasma alkylresorcinols as biomarkers of various stable WGW and WGR intake levels^58^ also in a population with a low consumption of rye. The results also suggest that the correlations between alkylresorcinols and the clinical markers observed in this study reflect the nature of these compounds as biomarkers rather than individual sources of bioactivity, which in the case of whole grains is likely attributed to multiple factors and may be subject to interindividual variability related to bioavailability and metabolism of the various whole-grain components.

Plasma mammalian lignan concentrations were not associated with the whole-grain intake likely due to their widespread distribution in plants. However, the linear dose-response trend between urinary enterolactone and total mammalian lignan concentrations/excretions and the WGR and WGW intake does suggest that urinary mammalian lignans reflect whole-grain intake at recommended levels. This parallels observations with flaxseed intake^59^, confirming urinary mammalian lignans reflect intake of lignan-rich foods but lack whole-grain specificity.

The study has several strengths and limitations. It was designed to investigate the usefulness of alkylresorcinols and mammalian lignans as biomarkers of whole-grain intake and to explore the use of targeted and untargeted metabolomics approaches to identify previously uncharacterized metabolites as alternative biomarkers of whole-grain intake. To our knowledge, this had not been tested in a (UK) population with a habitual low whole-grain intake based on wheat as the dominant cereal consumed. The number of subjects required for the study was based on predicted changes in alkylresorcinol concentrations from an earlier study^60^ and would not have been sufficient to see changes in lipid profile where a much larger sample size would be required^61^. The significant negative associations between plasma total alkylresorcinols concentrations and total and LDL cholesterol concentrations, especially in the WGR intervention, supports the lipid-lowering properties of rye, even though there were no significant effects of whole-grain intervention per se (i.e., treatment/time effects) on these markers. Unfortunately, we were not able to obtain blood samples at the Induction visit, so it was not possible to compare metabolite profiles of the participants consuming their habitual diet and after excluding whole grains. Whole-grain foods were provided for all volunteers; they were packaged to allow easy calculation of amounts required and the range of foods restricted to reduce variability in whole-grain intakes. Self-reported compliance to the dietary intervention was excellent and was confirmed by the positive associations between self-reported whole-grain intake and plasma alkylresorcinol concentrations as well as the different profile of alkylresorcinols homologs for the two intervention groups. Changes in metabolic profiles, therefore, can be attributed directly to the intervention foods. Limited conclusions can be made based on the effects of the WG diets on the gut microbiota due to the lack of metagenomics data from fecal samples.

An inherent limitation particularly in LC-MS-based metabolomics is the large proportion of unidentified features, which stems from the high sensitivity of the instrument producing thousands of signals from each sample and the limited availability of reference spectra from public or in-house databases. Potentially novel metabolites arising from the whole-grain intervention may still lack a reference in available databases, and they are more likely to fail any identification attempt. The inclusion of three time points in this study—baseline, endpoint of the first intervention, and endpoint of the second intervention—are not sufficient to confirm the linearity of the changes or to assess whether a steady state in the metabolite levels was achieved within each intervention period. Rather, the results should be considered directional in this regard.

In summary, we have found that the plasma metabolite profile and specific biomarkers of cardiometabolic risk of healthy adults were impacted by both whole-grain wheat and rye, especially at 96 grams of whole grain per day, with some common and some divergent effects of the two different cereal species. The decreased acylcarnitines and their associations with glycerol suggest further evidence of the cardioprotective effects of whole grains. We have also confirmed that alkylresorcinols are suitable and stable markers of whole-grain wheat and whole-grain rye intake and that a single blood collection is sufficient for estimating regular and frequent whole-grain intake under controlled intervention conditions. The identification of several gut microbial–host co-metabolites, including previously unreported candidates such as sinapyl alcohol, increasing with the intake of whole-grain rye suggests that the rye–gut microbiome is potentially more important as a mechanistic route in relation to health effects and underlines the importance of studying different cereal species individually.

## Methods

### Subjects

The volunteers for the GrainMark dietary intervention study were recruited from the Tyne & Wear region of the UK. The sample size was calculated using data on differences in alkylresorcinol concentrations in a study in which subjects consumed whole-grain rye in comparison with refined wheat^60^. The data contained measurements from baseline and wash-out periods and changes in alkylresorcinol concentration in whole-grain wheat (WGW) compared with whole-grain rye (WGR; 412 µg/g and 726 µg/g, respectively^27^) were estimated. Thus, a mean difference in plasma alkylresorcinol concentration in plasma from baseline of 97.7 ± 12.1 nmol/ml to a maximum of 352 ± 24.7 nmol/ml (mean ± SE) required 54 subjects providing 90% power to detect this difference at a significance level of *p* = 0.05. The inclusion criteria for the study were males or females having a BMI between 20–32 kg/m^2^ and above 40 years of age. The exclusion criteria for the study were smoking, allergies or intolerances to intervention foods, undergoing any clinical treatment and/or taking prescribed medications, using dietary supplements, having any dietary restrictions (except being a vegetarian), planning to change dietary habits, increased physical activity or changed body weight and being pregnant. The project was approved by the National Research Ethics Service (reference number 07/H0902/53). All volunteer-related procedures in this research were undertaken at the Newcastle Biomedicine Clinical Research Facility (CRF) under R & D approval reference no. 4349. The study is registered on the International Standard Randomized Controlled Trial Number Registry (ISRCTN), no. ISRCTN93493245.

In total, 70 volunteers were recruited into the study following pre-screening and 2 volunteers dropped out due to medical reasons not related to the study before randomization (Supplementary Fig. 8). All the participants completed and signed an informed consent form at the induction visit. There were 35 (17 M, 18 F) volunteers in the WGW group and 33 (16 M, 17 F) volunteers in the WGR group. The mean (SD) age of the volunteers at the start of the intervention was 55.0 (6.48) years and 54.2 (5.22) years in the WGW and WGR groups, respectively. The mean (SD) BMI of the volunteers at the start of the intervention was 25.9 (3.23) kg/m^2^ and 25.6 (3.22) kg/m^2^, for the WGW and WGR groups, respectively.

### Trial design

The study was a two-group parallel design randomized dietary intervention with a controlled increase in the intake of either WGW or WGR foods (Supplementary Fig. 9). In brief, after a 4-week ‘wash-out’ period when the volunteers avoided all whole-grain foods (Dose 0) they were asked to consume 3 servings of whole grains per day (approximately 48 g of whole grains per day) of either WGW or WGR (Dose 1) for the next 4 weeks. In the final phase (Dose 2) of the study, volunteers were asked to consume 6 servings per day of the same whole-grain foods they had during the Dose 1 period, for a further 4 weeks (equivalent to approximately 96 g of whole grains per day). Volunteers were randomly allocated to either of the intervention groups using random number tables by a study researcher. To aid compliance, the volunteers were provided with a list of common foods consumed in the UK that contain whole grains and were also given standardized verbal guidance on identifying whole-grain foods in general. Intervention foods for each group were provided by the research team and these are listed in Supplementary Table 4. All foods except WGW and WGR breads and Weetabix were packed individually in sealed foil containers with each packet containing pre-weighed amounts of different whole-grain foods based on the number of servings of whole grains present in them and were individually labeled to aid volunteer compliance. Each serving of whole-grain food approximated 16 g of whole grains based on the minimum serving size recommended by the USDA in its Dietary Guidelines for Americans 2010^17^. Volunteers were asked to continue to avoid the consumption of whole grains from any other sources of foods throughout the intervention period, unless specifically provided by the study team. The volunteers could attain the required dose of whole grains using any combination of foods that were provided to them.

### Sampling protocols and dietary monitoring

At the end of each intervention period (Dose 0, Dose 1, and Dose 2), volunteers visited the study facilities for biofluid sampling and anthropometric measurements on two separate occasions, following an overnight fast of at least 10 h before each occasion, placed two days apart in order to gauge biomarker variability. To reduce random intra-individual and inter-individual variations in blood/urine samples caused by the acute influence of the evening meal before sampling days, all volunteers were provided with the same set evening meal that was consumed by 8 pm the evening before every measurement visit. The meals were chosen to provide approximately 30% of daily energy and nutrient needs for an ‘average’ adult volunteer. The components of the meal were chosen so that they exerted minimal effects on the biological markers measured in the biofluid samples collected the following day, specifically ensuring that there were no whole-grain ingredients in them (Supplementary Table 5). Water intake was also controlled by the provision of a set amount of bottled water.

Blood samples were collected in lithium heparin vacutainers (BD Vacutainers, UK), and either sent immediately for lipid profile and glucose analysis or were inverted gently three times, placed on ice and then centrifuged within 2 h at 1200 × *g* for 10 min, at 4 °C. Following centrifugation, plasma samples were immediately stored at −80 °C until analyzed in batches at the end of the dietary intervention. The 24-h urine samples were collected in plastic containers without preservatives. Instead, the volunteers were provided with cool bags and ice packs to keep the urine samples cold throughout the collection period. The urine samples were aliquoted and stored at −80 °C until analyzed. Seated blood pressure was measured using an Omron Intellisense Automatic BP Monitor (Omron Healthcare UK Ltd., Milton Keynes, UK).

A validated 7-day Food Frequency Questionnaire (FFQ)^61^ was used to assess dietary intake at the induction visit (representing habitual diet), and subsequently in the middle of each 4-week intervention period (Dose 0, Dose 1, Dose 2). The FFQ was used to assess whole-grain food intake as well as to estimate the overall energy and nutrient intake during various intervention phases, using an established nutrient database based on McCance & Widdowson 6th Edition food tables^62^. The volunteers also completed ‘intervention food records’ to record the type and frequency of the different whole-grain intervention foods consumed during Dose 1 and Dose 2 periods, which also served to help volunteers achieve the required amounts of either WGR or WGW intervention foods in their daily diet and were subsequently used as compliance checks for the dietary intervention as well as to assess the actual intervention whole-grain foods selected from the whole-grain foods provided. The average daily alkylresorcinol intake from the study whole-grain foods was calculated for each dietary intervention period using the food record data and the alkylresorcinol content of the study foods (Supplementary Table 6) provided to the volunteers in the two whole-grain groups^34^.

### Analytical methods

#### Plasma lipids and other clinical markers

Plasma lipid profile and glucose were analyzed on fresh samples by standard clinical chemistry procedures by the Clinical Biochemistry Department of the Newcastle upon Tyne NHS Foundation Trust. E-selectin, Intercellular Adhesion Molecule-1 (ICAM-1) and vascular cell adhesion molecule-1 (VCAM-1) concentrations in plasma were analyzed by ELISA assays using DuoSet® ELISA Development System kits (R&D Systems Europe Ltd., Abingdon, Oxfordshire, UK). Insulin resistance was calculated using the HOMA-IR (Homeostasis model assessment for insulin resistance) method^63^.

#### Alkylresorcinols and mammalian lignans

Plasma alkylresorcinols were measured by gas-chromatography-mass spectrometry (GC-MS)^64^ after sample derivatization with Methylsilyltetrafluroacetate plus 1% trimethylchlorosilane (MSTFA + 1% TMCS; Thermo Fisher Scientific, UK) for 30 min. The silylated samples were then quantified by GC-MS using a TR-5 column (15 m × 0.25 mm × 0.25 μm, Thermo Fisher Scientific, UK) on a Shimadzu QP-2010 (Shimadzu UK Ltd, Milton Keynes, UK). The detection of alkylresorcinols was based on the peak areas of their molecular ions^64^ using synthetic alkylresorcinol (AR) homologs (AR C17:0, AR C19:0, AR C20:0, AR C21:0, AR C23:0 and AR C25:0; all from ReseaChem AG, Burgsdorf, Switzerland) to generate standard curves and check retention time accuracy. Plasma and urinary concentrations of the mammalian lignans enterodiol and enterolactone were measured by high-performance liquid chromatography (HPLC) with Coularray electrochemical detection (ECD) using methods previously published^65,66^.

#### Plasma metabolome

Plasma samples were prepared for LC-MS according to ref. ^67^. Briefly, the frozen samples were thawed on iced water and kept on wet ice during all waiting periods. A 96-well filter plate was placed on the ice and 400 µl of cold ACN was added to each well. The plasma samples were vortexed for 10 s at full speed, and 100 µl of the vortexed sample was added to each well. In addition, 10 µl aliquots were collected from each sample into a separate microcentrifuge tube to create the quality control (QC) sample. The ACN and sample material were mixed inside each well by pipetting four times using a wide-orifice tip. The samples were centrifuged in the plate for 5 min at 700 × *g* and 4 °C to produce a clear supernatant for analysis and sealed immediately with a 96-well cap mat. The samples were stored at −20 °C until the LC-MS analysis.

#### LC-MS analysis

The untargeted LC-MS metabolomics analysis was performed according to Klåvus et al. (2020) and Hanhineva et al.^31,67^. Briefly, the samples were analyzed using a UPLC–QTOF–MS system (Agilent Technologies, Santa Clara, CA, USA), which consisted of a 1290 LC system, a Jetstream electrospray ionization (ESI) source, and a 6540 UHD QTOF (quadrupole-time-of-flight) mass spectrometer. The samples were separated using both reversed-phase liquid chromatography (RPLC; Zorbax Eclipse XDB-C18, particle size 1.8 µm, 2.1 × 100 mm; Agilent Technologies) and hydrophilic interaction liquid chromatography (HILIC; Aqcuity UPLC BEH amide, 2.1 × 100 mm, 1.7 µm; Waters Corp., Milford, MA, USA), and data were acquired with both positive and negative polarity, thus yielding four runs for each sample. QC samples were injected at the beginning and at the end of the LC-MS run and after every ten injections. Automatic data-dependent MS/MS analysis was performed on samples representing each sample type and on the QC samples. The sample tray was kept at +4 °C during the analysis.

#### Diet and plasma alkylresorcinol data analysis

The dietary intake at various stages of dietary intervention in WGW and WGR groups was compared using analysis of variance (ANOVA) and Tukey’s honestly significant difference test. A linear mixed-effects model (LMEM) was used to investigate the effects of whole-grain intake at different doses on plasma alkylresorcinol concentrations, adjusting for age, gender and BMI as covariates. The possibility of covariates having a fixed effect on these dependent measurements, whilst simultaneously allowing both a fixed and random effect for the whole-grain dose was investigated. The fixed effect for dose models the overall slope (if any) of the dependent variable through different doses of whole-grain intake; the random effect for dose models the variability about this overall slope, allowing different subjects in the study to have different dose responses. The linear mixed effects model analyses were performed using the statistical package R^68^. The same program was used to check the goodness-of-fit of the models using standard regression diagnostic tools, and to perform non-parametric tests on non-normally distributed data. The same LMEMs were used to investigate the associations of markers of cardiometabolic risk (e.g., plasma glucose, cholesterol, blood pressure, etc.) with either plasma alkylresorcinols or plasma mammalian lignans, or urinary mammalian lignans or mean whole-grain intake, after adjusting for age, gender and ‘cohort’ as covariates. For management purposes, volunteers were recruited in two cohorts over two 4-month periods. Therefore, in order to adjust for any seasonal influence in response to whole-grain intervention cohort effects, where significant, were adjusted for in the LMEMs. Pearson correlations were used to check for any significant dose-response associations between whole-grain intake and biomarker concentrations in plasma/urine. Due to skewed plasma alkylresorcinol data, Mann–Whitney tests were used to compare differences between different plasma samples taken at the same time period. Intra-class correlation coefficients were measured using Pearson’s product moment correlation coefficient, the significance of which were checked by referring our estimates to critical values from Student’s *t* distribution.

#### LC-MS metabolomics data analysis

The signal detection from raw LC-MS data was performed with MS-DIAL version 3.00 and the data pre-processing in R version 3.5.1 as described previously^67^. The detected features were corrected for drift caused by the LC-MS procedure and assessed for their quality, and low-quality features were flagged based on data from the QC samples. Features were kept if their RSD* was below 20% and their D-ratio below 40%. In addition, features with classic RSD, RSD* and basic D-ratio all below 10% were kept to prevent the flagging of features with very low values in all but a few samples. Missing values were imputed using random forest imputation. The four analytical modes were then combined before statistical analysis. The number of detected molecular features in the HILIC− mode was 3928, out of which 2898 were flagged and 1030 kept; in the HILIC+ mode, out of 1641 molecular features, 606 were flagged and 1035 kept; in the RP− mode, out of 3722 molecular features, 2303 were flagged and 1419 kept; and in the RP+ mode, out of 12 471 molecular features, 9326 were flagged and 3145 kept. Thus, out of total 21 762 molecular features, 15 133 were flagged and 6629 kept for the final data matrix.

Linear mixed models were performed separately for each of the 6629 ‘good-quality’ molecular features using R packages lme4 (version 1.1)^69^ and lmerTest (version 3.1.2)^70^. First, linear mixed models with time, intervention group and their interaction as fixed effects were fit for each feature. Then, changes in each intervention group were analyzed separately with a linear mixed model with the study stage as the fixed effect. Feature level was used as the dependent variable and subject identifier as a random effect in both models. The reported estimates are regression coefficients of the models. For the interaction terms, this is approximately the difference between the mean change in rye group and mean change in wheat group (if estimate is positive, rye went up compared to wheat). For the analysis done separately on both groups, the regression coefficients approximate the change in the normalized mean levels at each time point and are expressed as z-normalized values. The *p*-values of all statistical tests were corrected with Benjamini–Hochberg false discovery rate (FDR). The Spearman correlations between the metabolomics data and clinical markers were calculated with notame. The adjusted *p*-value (*q*-value) *q* < 0.1 was considered significant. We deviated from the traditional threshold of 0.05 because of the exploratory and hypothesis-generating nature of the study.

#### Metabolite annotation

Significantly differential molecular features were manually annotated based on an in-house library of reference standards containing MS/MS spectra and retention times of 602 metabolites, a database file compiled of publicly available MS/MS spectra, including MassBank, MoNA, ReSpect phytochemical database, and GNPS^71^, as well as MS/MS fragmentation patterns from known biochemicals generated in silico in MS-FINDER software^28^. The metabolites were assigned an identification level to allow assessment of annotation reliability according to ref. ^72^: level 1 was given for identifications based on the in-house database, level 2 for putative annotations based on publicly available spectral databases, level 3 for putative characterizations based on spectral similarity to known metabolite classes, and level 4 for all the remaining unknown features. The observed LC-MS characteristics and statistical results of the annotated metabolites are presented in Supplementary Table 7.

### Reporting summary

Further information on research design is available in the Nature Research Reporting Summary linked to this article.

### Supplementary information

Reporting summary

Supplemental material

Supplementary Table 7

## Data Availability

Data described in the manuscript is publicly and freely available without restriction at EUDAT B2SHARE [https://b2share.eudat.eu/records/66c289b7af8f4b96bafdc906e17ce9eb].

## Code availability

The code used for the untargeted metabolomics data is available at https://github.com/antonvsdata/notame.
