# Supplementary material for: Metabolic changes in response to varying whole-grain wheat and rye intake
Source: NPJ Sci Food. 2024 Jan 30;8:8. doi: 10.1038/s41538-024-00247-0 (PMC10828387; doi:10.1038/s41538-024-00247-0)
Supplement: Supplementary file 2 — Supplemental material [file 41538_2024_247_MOESM2_ESM.pdf]

Supplementary Material

Supplementary Figure 1

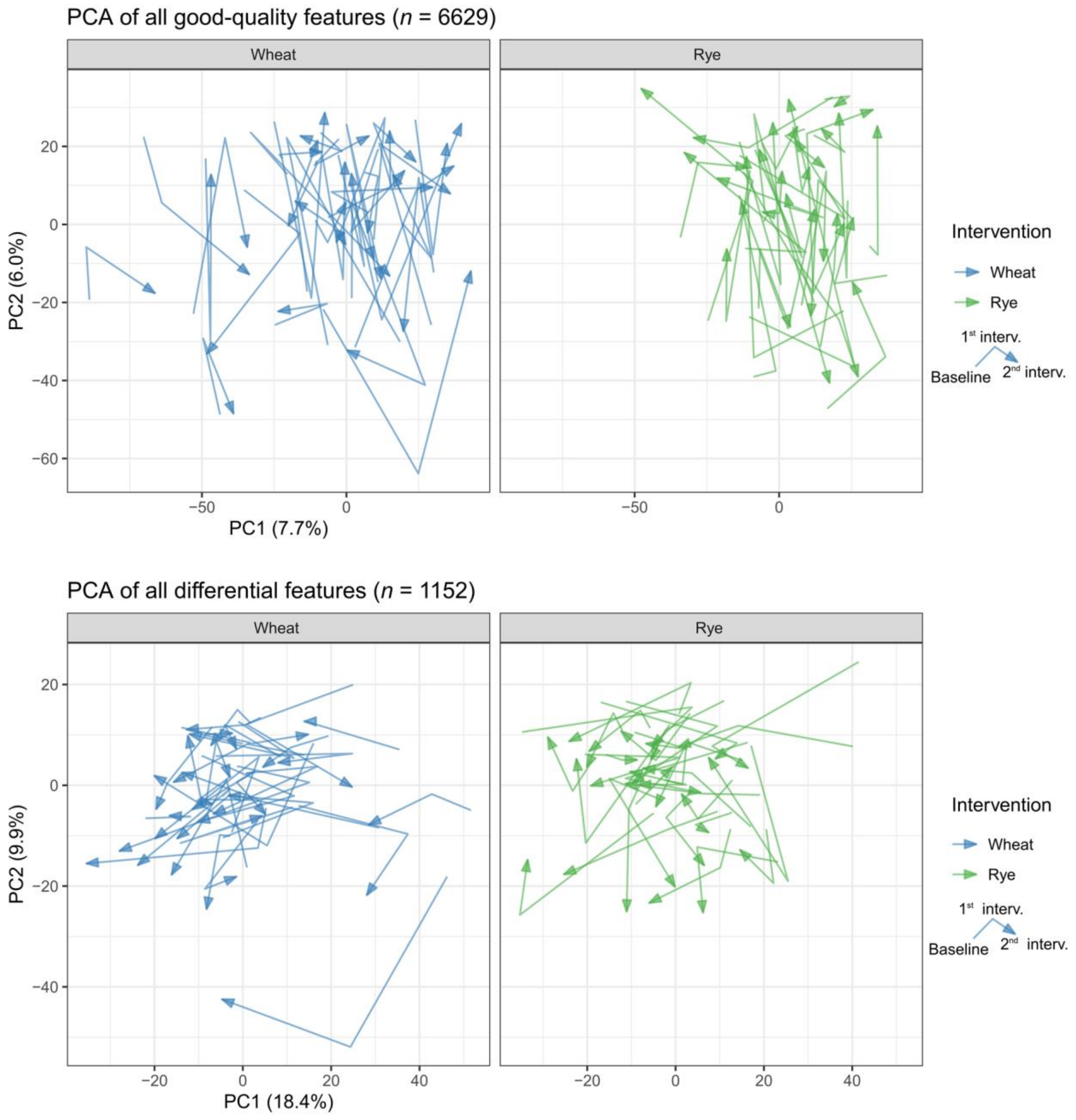

Supplementary Figure 1. Principal component analysis (PCA) of the dataset based on the levels of all the good-quality features after drift correction (*n* = 6629) and all the differential good-quality features in the statistical comparisons (*n* = 1152) from the four analytical modes, divided into two identical scale figures based on the intervention group. The arrows represent the changes of the metabolite profiles of each subject from baseline (beginning of arrow) to the second intervention (arrowhead). The pooled QC samples (*n* = 19) were used to control and validate the uniformity of the data throughout the analytical runs.

Supplementary Figure 2

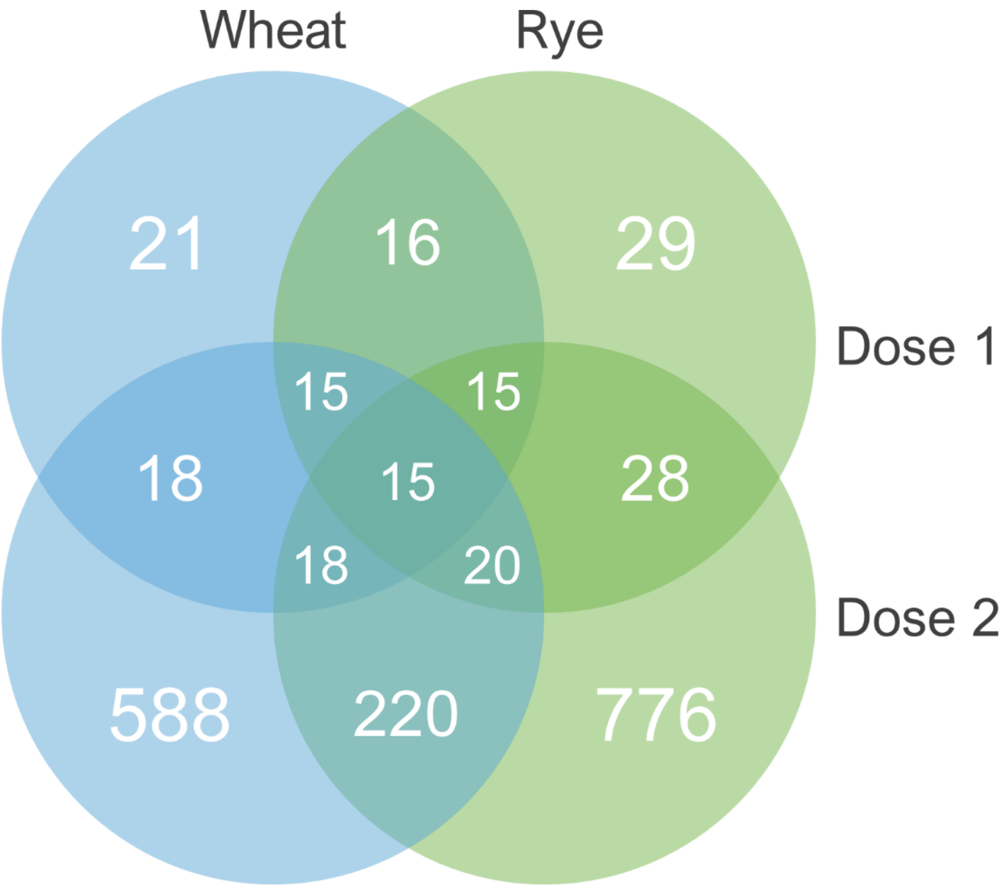

Supplementary Figure 2. Euler diagram of the statistically significant ($q < 0.1$) good-quality molecular features in each grain/dose group compared to baseline.

Supplementary Figure 3

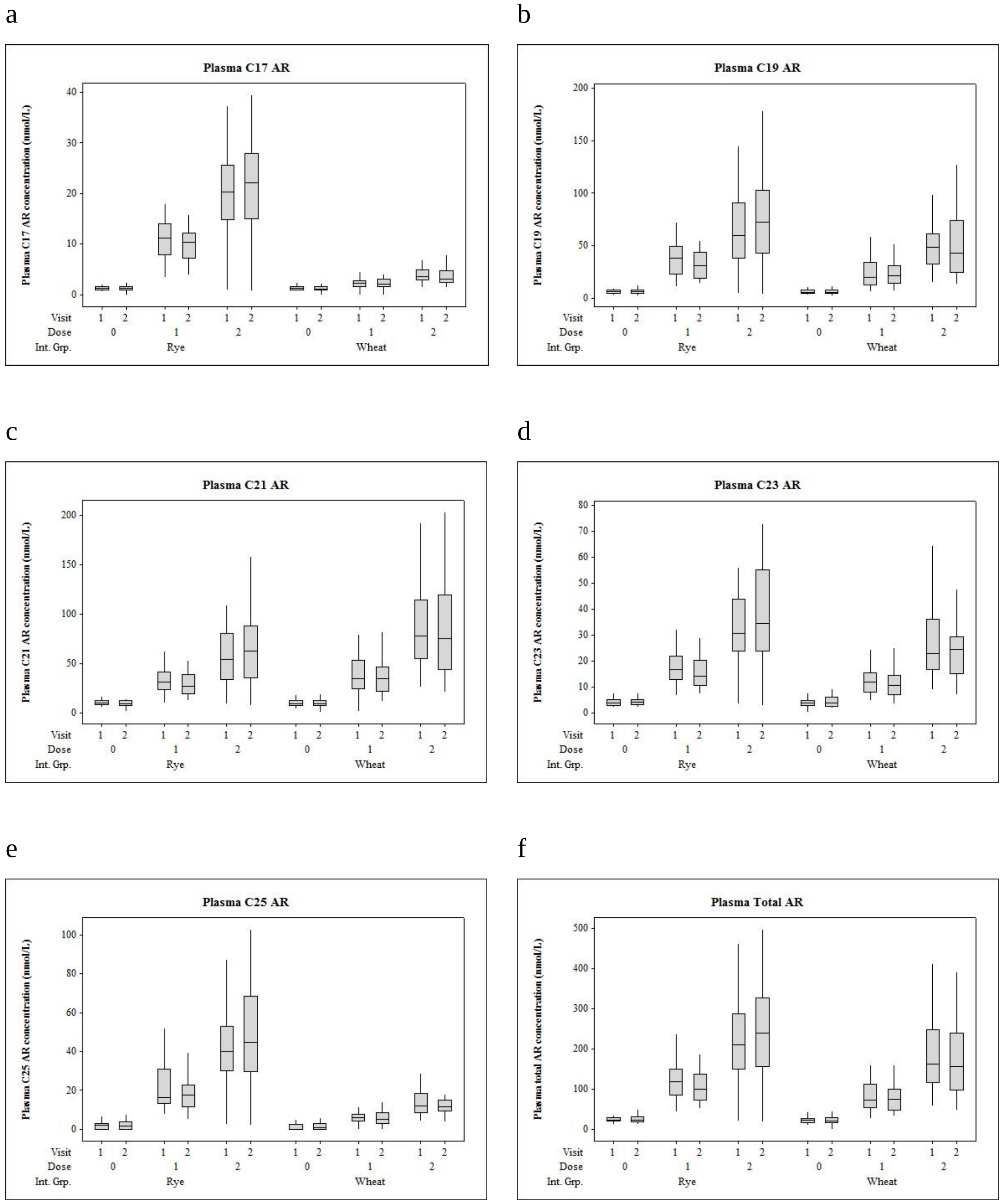

Supplementary Figure 3. Changes in individual (a, C17; b, C19; c, C21; d, C23; e, C25) and total (f) plasma AR homologue concentrations with increasing intake of WGR or WGW. Visit 1 and Visit 2 are repeated plasma samples taken two days apart at the end of each four-week intervention period. Box plots represent median values and interquartile range.

Supplementary Figure 4

a

b

c

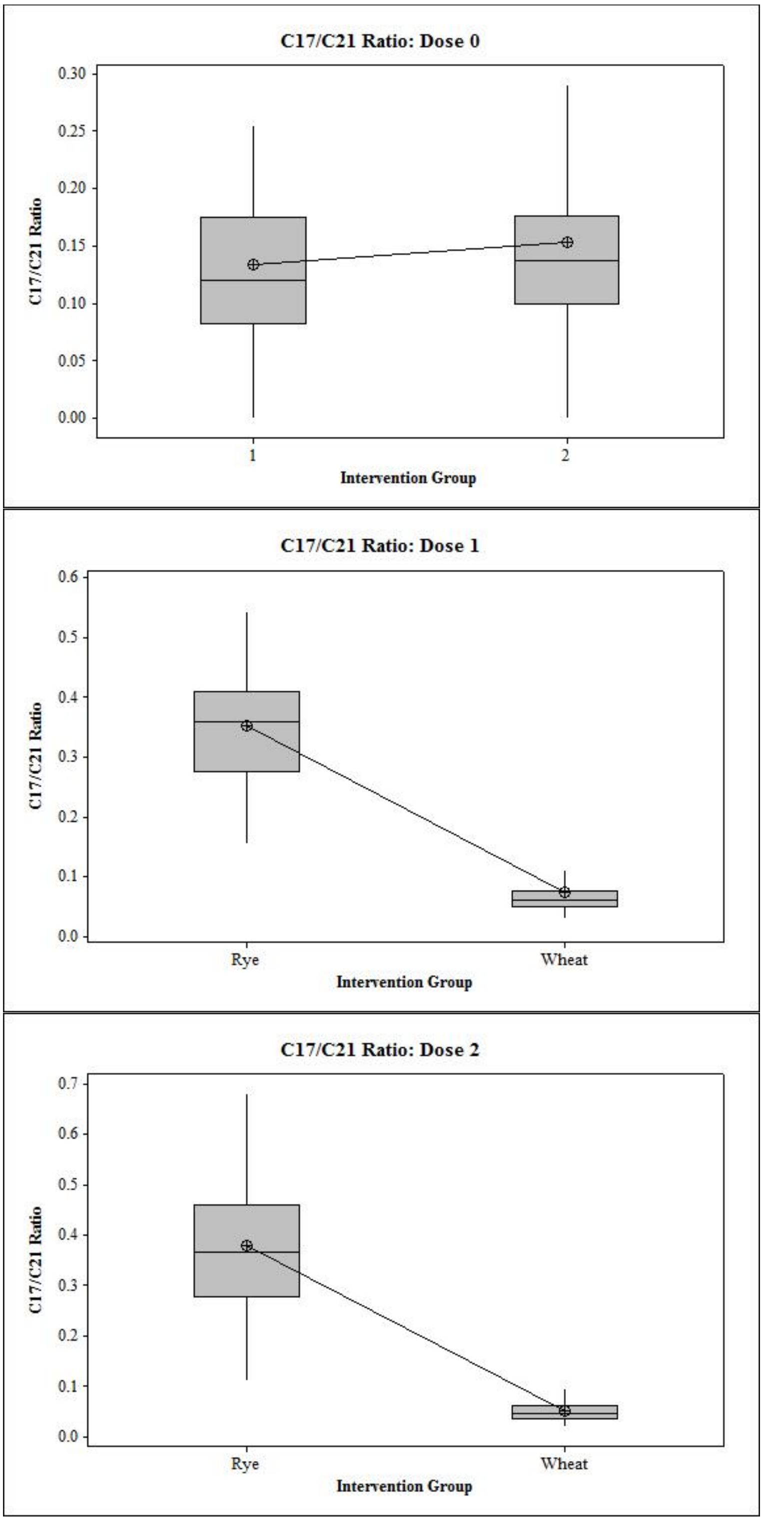

Supplementary Figure 4. Plasma C17:0 to C21:0 AR homologue ratio for WGR and WGW groups at the end of each dose of dietary intervention. Box plots represent median values and interquartile range; circles denote mean values.

Supplementary Figure 5

a

b

c

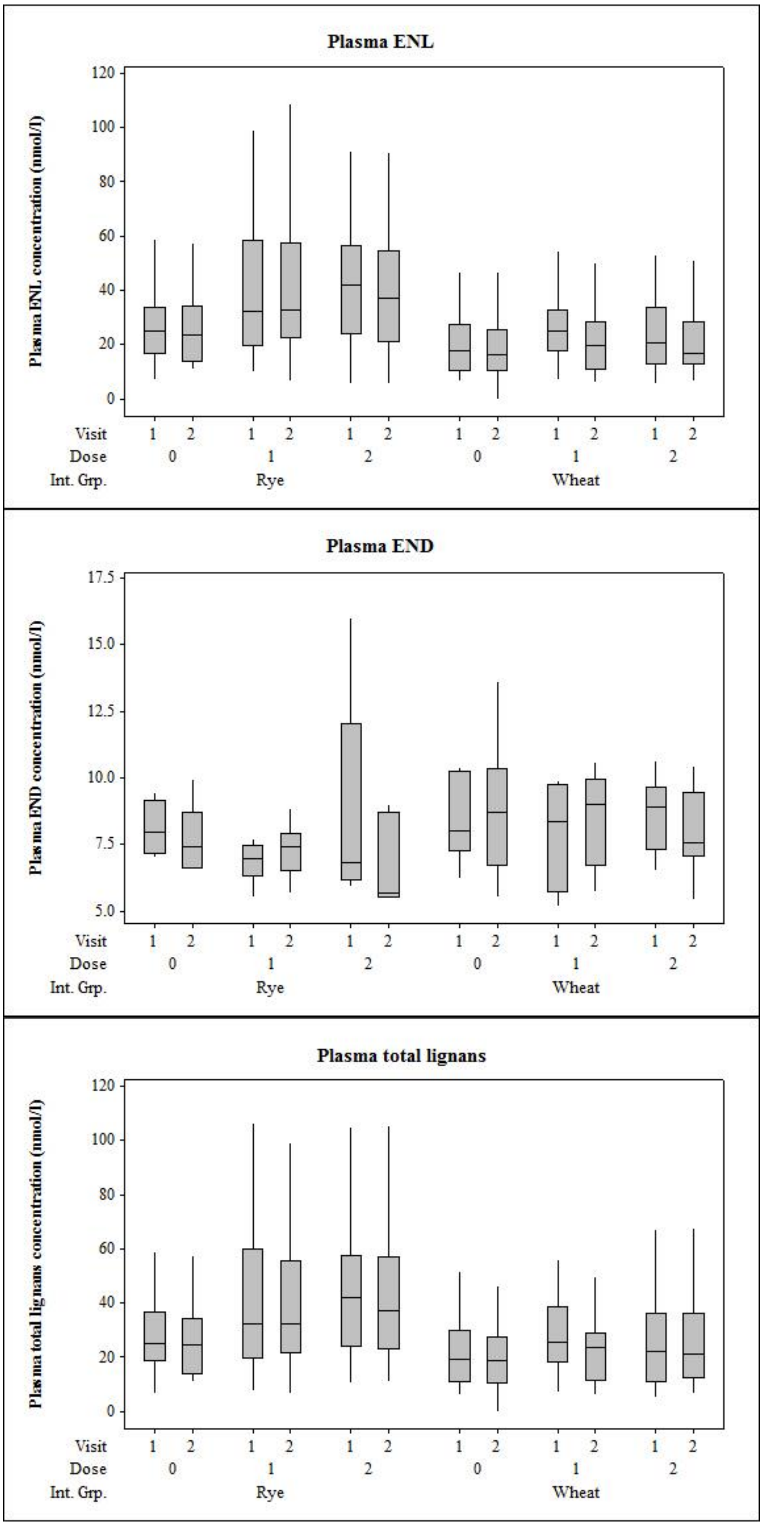

Supplementary Figure 5. Changes in plasma concentrations of enterolactone (A, ENL), enterodiol (B, END) and total ML (C) with increasing intake of WGR or WGW. Visit 1 and Visit 2 are repeated plasma samples taken two days apart at the end of each four-week intervention period. Box plots represent median values and interquartile range.

Supplementary Figure 6

a

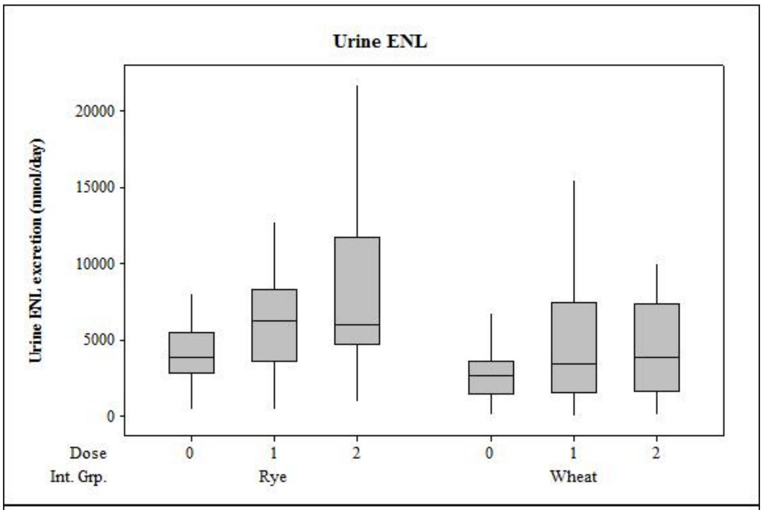

b

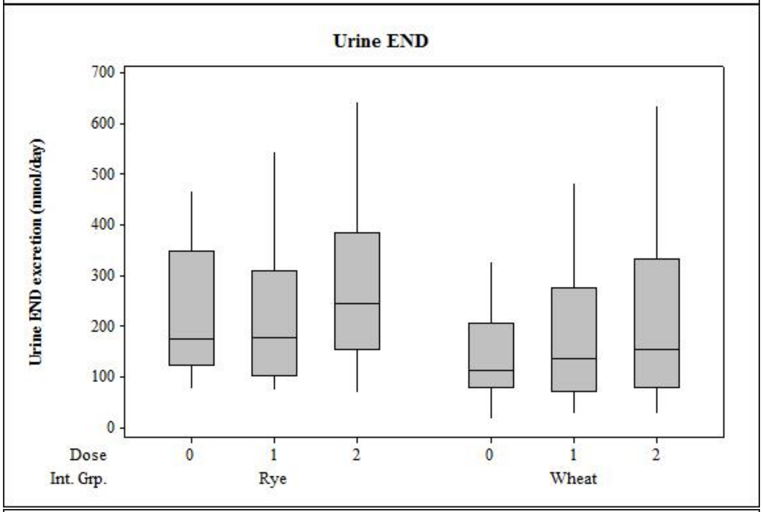

c

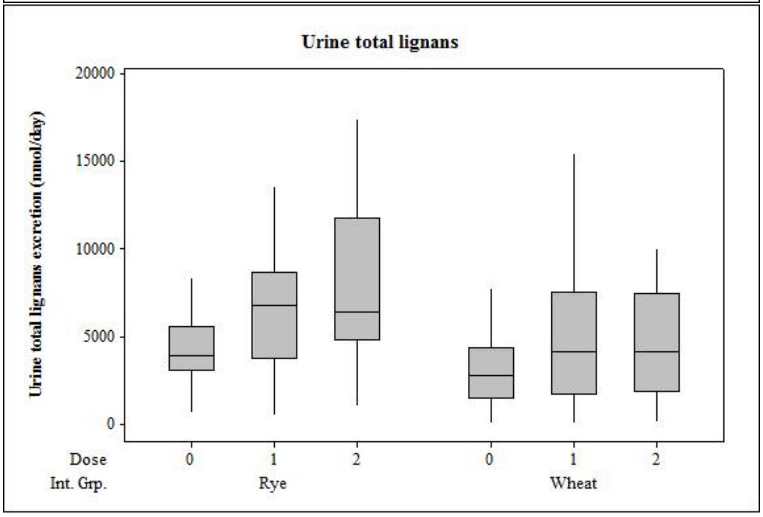

Supplementary Figure 6. Changes in daily urinary excretions of enterolactone (A, ENL), enterodiol (B, END) and total ML (C) with increasing intake of WGR or WGW. Box plots represent median values and interquartile range.

Supplementary Figure 7

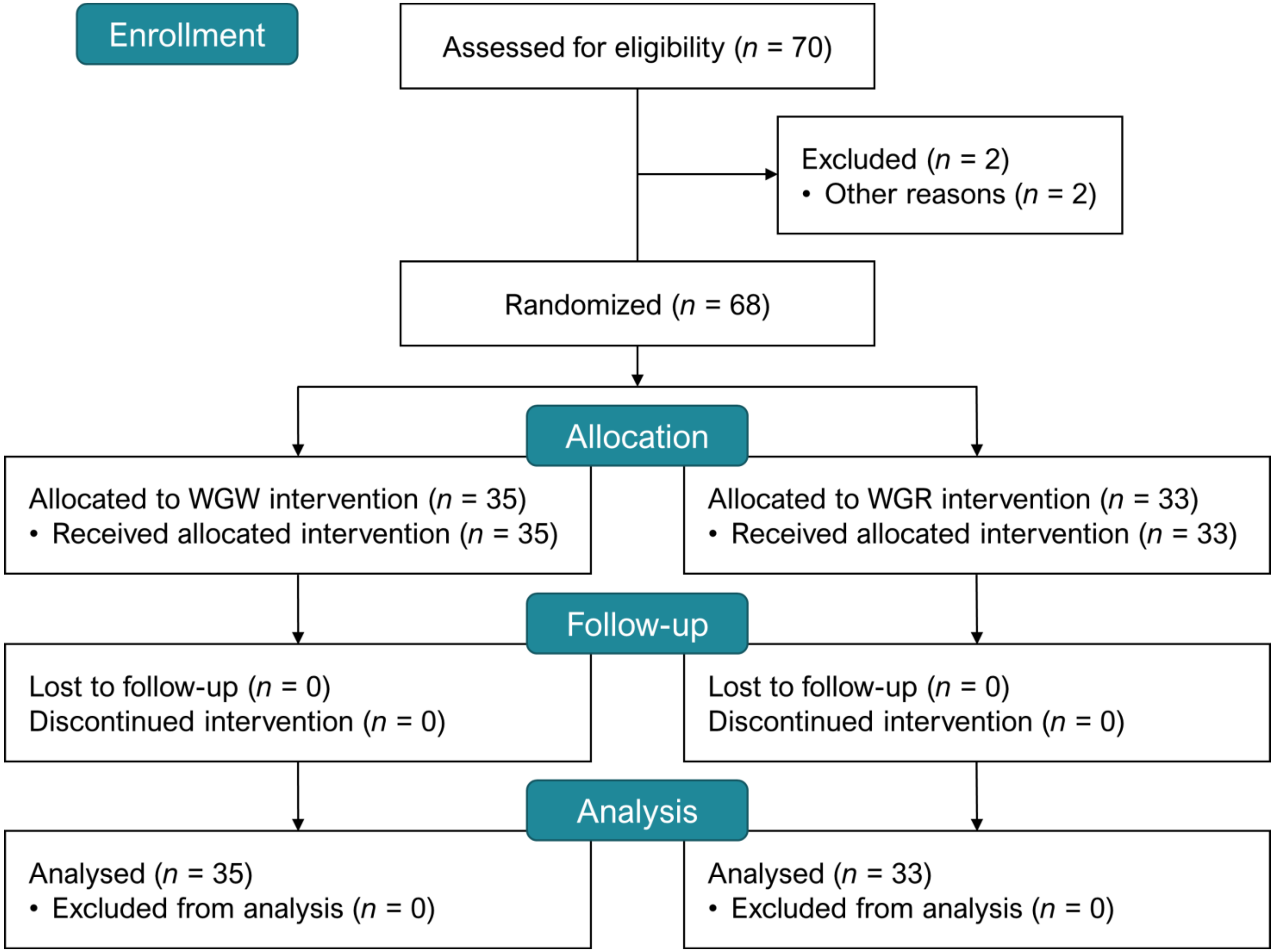

Supplementary Figure 7. The participant flowchart.

Supplementary Figure 8

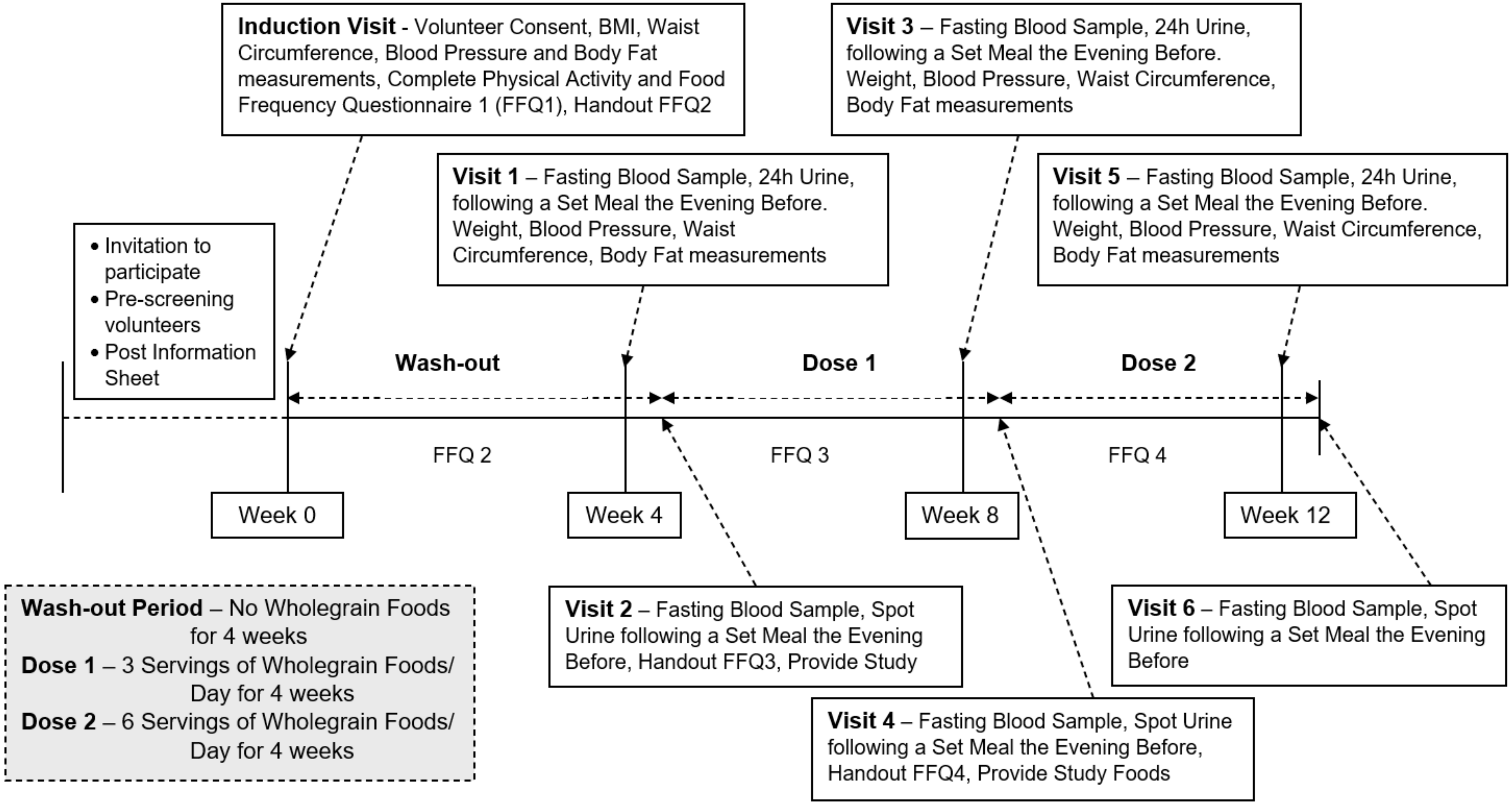

Supplementary Figure 8. The GrainMark study design flowchart.

Supplementary Table 1. Intervention food selection provided during the study.

	Whole-Grain Wheat Group								Whole-Grain Rye Group							
Mean daily intake	Induction Visit		Wash-out		Dose 1		Dose 2		Induction Visit		Wash-out		Dose 1		Dose 2	
	Mean	SD	Mean	SD	Mean	SD	Mean	SD	Mean	SD	Mean	SD	Mean	SD	Mean	SD
Energy (MJ)	12.19	0.39	11.87	0.48	11.72	0.36	12.93	0.36	12.59	0.42	12.38	0.35	12.98	0.38	14.12	0.40
Carbohydrate (g)	368.1	140.0	353.6	174.3	350.3	119.3	408.9	120.8	376.1	127.3	369.2	118.4	374.9	105.3	450.7	**141.2**^{A}
Total fat (g)	90.9	33.6	85.2	43.7	80.8	31.3	88.7	32.5	96.4	45.3	91.3	36.0	97.0	32.1	100.6	**39.1**^{aA}
- SFA (g)	26.6	10.7	27.1	15.2	25.4	12.1	27.0	12.6	26.9	13.3	27.6	13.4	27.9	10.1	27.7	12.7
- PUFA (g)	8.8	2.9	9.1	4.1	8.6	3.6	9.5	4.0	10.6	4.2	10.1	3.8	11.1	4.1	11.0	3.5
- MUFA (g)	21.1	7.1	22.3	11.7	20.6	8.9	22.6	10.3	23.6	10.5	23.7	9.4	24.7	8.21	23.1	9.1
Protein (g)	100.0	32.1	98.4	35.9	97.0	28.4	108.8	31.6	105.5	28.3	97.6	28.8	101.2	26.0	111.2	28.9
Alcohol (g)	41.6	38.2	46.8	43.3	50.2	55.9	42.5	47.9	40.9	33.1	48.6	41.4	56.8	64.6	44.2	33.7
Fibre (g)†	28.3	12.0	18.2	**6.7**^{C}	24.3	**7.1**^{A}	36.0	**10.7**^{cC}	27.9	8.5	20.1	**6.0**^{c}	23.3	6.2	30.5	**9.2**^{C}
Folate (µg)	402.9	132.0	375.5	125.4	371.9	146.7	431.5	125.6	411.6	125.9	383.0	109.9	351.9	105.6	378.0	121.4
Vitamin B6 (mg)	2.8	0.9	2.8	0.9	2.8	0.9	3.2	0.9	2.9	0.8	2.9	0.7	2.7	0.7	2.8	0.8
Thiamin (mg)	2.3	0.9	2.0	0.8	2.2	0.7	2.9	**0.8**^{bC}	2.4	0.6	2.0	0.5	2.1	0.5	2.5	**0.7**^{A}
Riboflavin (mg)	2.1	0.7	2.0	0.8	2.3	0.7	2.9	**0.7**^{cC}	2.1	0.7	2.0	0.6	1.9	0.5	2.1	0.6
Vitamin C (mg)	159.4	64.5	146.8	57.9	144.6	72.2	140.2	64.3	159.3	76.6	153.3	63.7	139.9	49.9	147.0	56.9
Vitamin E (mg)	7.9	2.9	6.6	2.3	6.6	2.1	7.4	2.1	8.7	3.1	7.5	2.5	8.8	2.8	11.1	**2.9**^{bC}
Sodium (g)	4.1	1.6	4.0	1.9	3.6	1.2	4.3	1.5	4.4	1.4	4.1	1.3	4.3	1.5	5.1	**1.7**^{a}
Potassium (g)	4.8	1.3	4.4	1.3	4.4	1.1	4.8	1.3	4.9	1.4	4.5	1.0	4.7	1.2	5.0	1.2
Calcium (mg)	955.7	298.9	978.3	504.8	875.5	335.6	854.4	261.8	978.6	299.9	952.7	306.0	1008.0	327.9	1127.9	342.3
Iron (mg)	20.3	8.3	17.0	6.3	19.2	6.8	24.2	**6.4**^{C}	20.9	5.8	17.9	4.7	19.6	6.1	22.2	**5.9**^{A}
Manganese (mg)	7.6	3.3	4.9	**1.6**^{c}	5.9	**2.0**^{a}	8.5	**2.9**^{C}	7.7	3.0	5.2	1.6	6.3	1.9	8.1	**2.7**^{C}
Magnesium (mg)	489.8	175.9	369.6	**124.9**^{b}	427.3	117.8	537.8	**142.5**^{C}	508.7	150.0	390.9	93.2	457.8	128.7	538.5	**137.3**^{C}
Phosphorus (g)	1.8	0.6	1.5	0.6	1.6	0.5	2.0	**0.6**^{B}	1.8	0.5	1.5	0.4	1.7	0.4	2.0	**0.5**^{C}
Zinc (mg)	12.7	4.9	10.2	3.9	11.0	3.4	13.8	**4.2**^{B}	12.6	3.8	10.0	2.9	11.5	3.1	13.5	**3.5**^{C}

†Englyst fiber

Values in bold that are significantly different from corresponding Induction Week value: $^{a}p < 0.05$; $^{b}p < 0.01$; $^{c}p < 0.001$. Values in bold that are significantly different from corresponding Wash-out value: $^{A}p < 0.05$; $^{B}P < 0.01$; $^{C}p < 0.001$.

Supplementary Table 2. Pre-sampling evening meal.

Mean daily intake (mg/d)	WGW group				WGR group			
	Dose 1		Dose 2		Dose 1		Dose 2	
	Mean	SD	Mean	SD	Mean	SD	Mean	SD
C17 alkylresorcinol	1.4	0.23	2.9	0.41	11.0	0.99	23.6	2.58
C19 alkylresorcinol	9.6	1.92	20.9	3.37	13.1	1.46	28.6	3.78
C21 alkylresorcinol	16.0	2.81	34.7	4.59	12.1	1.29	26.3	3.32
C23 alkylresorcinol	4.5	0.82	9.8	1.19	7.4	0.77	16.1	1.99
C25 alkylresorcinol	1.8	0.35	3.8	0.51	6.3	0.69	13.7	1.78
Total alkylresorcinol	33.2	5.97	72.1	9.86	49.9	5.19	108.3	13.40
C17 to C21 intake ratio	0.1	0.01	0.1	0.01	0.9	0.02	0.9	0.03

Supplementary Table 3. Reported total WG intake for WG wheat (WGW) and WG rye (WGR) groups during the study, using Food Frequency Questionnaires (FFQ). Wash-out (Dose 0) was a 4 wk whole grain avoidance diet, Dose 1 required 48 g/d whole grain intake for 4 weeks and Dose 2 required 96 g/d whole grain intake for a further 4 weeks. Mean daily intakes of separate alkylresorcinol homologues for whole grain wheat and whole grain rye groups and AR C17: AR C21 intake ratios calculated from AR content of intervention foods and self-reported intervention food intake using food records.

	WGW Group					WGR Group				
Biomarker	Slope (ln)[a]	R	*P* value	Constant	Regression equation	Slope (ln)	R	*P* value	Constant	Regression equation
Plasma										
ENL	-[*]	-	-	-	-	+0.002	0.141	0.0301	5.076	5.08 + 0.002WG
END	-	-	-	-	-	-	-	-	-	-
Total ML	-	-	-	-	-	-	-	-	-	-
24h Urine						-	-	-	-	-
ENL	+0.003	0.197	0.0048	+0.003	4.17 + 0.003 WG	+0.003	0.214	0.0004	8.304	8.30 + 0.003WG
END	-	-	-	-	-	-	-	-	-	-
Total ML	+0.003	0.210	0.0031	+0.003	4.31 + 0.003 WG	+0.003	0.207	0.0005	8.388	8.39 + 0.003WG

[a]Natural logarithm; *Not detected

Supplementary Table 4. Mean and standard deviation (SD) of daily energy and nutrient intake during each stage of the intervention study.

	Supplier	Processing	Portion size (g)	WG (g per portion)	WG (serves per portion)	Alkylresorcinol (AR) homologues (%)					Total ARs
						C17:0	C19:0	C21:0	C23:0	C25:0	(μg/g)
Whole-grain rye diet											
Rye bread (50 % WG rye flour, 50 % sifted rye flour (40 % of bran removed))	Village Bakery, Penrith, UK	Sourdough fermentation and baking	25	20	1	17.9	23.3	20.9	12.6	11.0	752.7
Rye porridge (ready-to-eat cereal)	Raisio, Finland	Steamed and then rolled	35	32	2	19.5	22.0	20.4	12.5	10.5	877.7
Rye muesli (ready-to-eat cereal)	Raisio, Finland/Cereal Partners UK	Steamed and then rolled	55	34	2	19.5	22.0	20.4	12.5	10.5	877.7
Rye pasta (20 % WG rye, 80 % refined wheat)	Raisio, Finland	Extrusion, cooked by boiling	90	18	1	15.6	19.7	21.3	13.8	10.2	331.1
Whole-grain wheat diet											
Whole-grain wheat bread	Allied Bakeries, Gateshead, UK	Yeast fermentation and baking	36	20	1	3.4	26.7	41.7	11.3	4.6	499.9
Shredded Wheat Fruitful (RTE cereal)	Cereal Partners UK	Pressure cooked, flaked and baked	55	32	2	4.3	25.8	43.0	11.6	4.0	422.7
Weetabix (RTE cereal)	Weetabix, UK	Pressure cooked, flaked and baked	19	15	1	4.3	26.6	43.5	11.9	4.9	547.7
Whole-grain wheat pasta	Cereal Partners UK	Extrusion, cooked by boiling	54	48	3	0.8	11.4	47.8	22.7	8.6	477.0

Supplementary Table 5. Mean daily intakes of separate alkylresorcinol homologues for the WGW and WGR groups and AR C17 to AR C21 intake ratios during Dose 1 and Dose 2 periods as calculated from the alkylresorcinol content of intervention foods and self-reported intervention food intake using intervention food records.

Meal Type	Quantity	Major Ingredients	Manufacturer
Main Course – 'Chicken in a pot'	1 portion, 450 g	Roasted Chicken, Roast Potatoes, Mixed Vegetables in Gravy Sauce	Sainsbury's Supermarkets, UK
Desert – Chocolate Éclair	1 piece, 75g	Whipping cream, sugar, egg, wheat flour, cocoa	Marks and Spencer Plc, UK
Water	500 ml compulsory with meal + 500 ml optional overnight	Mineral Water	Sainsbury's Supermarkets, UK

Supplementary Table 6. Dose-response trends between WG intake against measured plasma and 24-hour urinary ENL, END and total ML concentrations in WGW and WGR groups, using linear mixed effect models, adjusted for age, gender and BMI. The slope is the mixed effect of the WG in g/day.

	WGW group				WGR group			
	Dose 1		Dose 2		Dose 1		Dose 2	
Mean daily intake (mg/d)	Mean	St. Dev.	Mean	St. Dev.	Mean	St. Dev.	Mean	St. Dev.
C17 alkylresorcinol	1.4	0.23	2.9	0.41	11.0	0.99	23.6	2.58
C19 alkylresorcinol	9.6	1.92	20.9	3.37	13.1	1.46	28.6	3.78
C21 alkylresorcinol	16.0	2.81	34.7	4.59	12.1	1.29	26.3	3.32
C23 alkylresorcinol	4.5	0.82	9.8	1.19	7.4	0.77	16.1	1.99
C25 alkylresorcinol	1.8	0.35	3.8	0.51	6.3	0.69	13.7	1.78
Total alkylresorcinol	33.2	5.97	72.1	9.86	49.9	5.19	108.3	13.40
C17 to C21 intake ratio	0.1	0.01	0.1	0.01	0.9	0.02	0.9	0.03
